# Environmental exposure disparities in ultrafine particles and PM_2.5_ by urbanicity and socio-demographics in New York state, 2013–2020

**DOI:** 10.1016/j.envres.2023.117246

**Published:** 2023-10-06

**Authors:** Arshad Arjunan Nair, Shao Lin, Gan Luo, Ian Ryan, Quan Qi, Xinlei Deng, Fangqun Yu

**Affiliations:** a Atmospheric Sciences Research Center, University at Albany, State University of New York, Albany, NY 12226, USA; b Department of Environmental Health Sciences, University at Albany, State University of New York, Rensselaer, NY 12144, USA; c Department of Epidemiology and Biostatistics, University at Albany, State University of New York, Rensselaer, NY 12144, USA; d Department of Economics, University at Albany, State University of New York, Albany, NY 12222, USA

**Keywords:** Air quality, Aerosols, Fine particulate matter, Ultrafine particles, Environmental justice, Public health inequalities

## Abstract

**Background:**

The spatiotemporal and demographic disparities in exposure to ultrafine particles (UFP; number concentrations of particulate matter (PM) with diameter ≤0.1 μm), a key subcomponent of fine aerosols (PM_2.5_; mass concentrations of PM ≤ 2.5 μm), have not been well studied.

**Objective:**

To quantify and compare the aerosol pollutant exposure disparities for UFP and PM_2.5_ by socio-demographic factors in New York State (NYS).

**Methods:**

Ambient atmospheric UFP and PM_2.5_ were quantified using a global three-dimensional model of chemical transport with state-of-the-science aerosol microphysical processes validated extensively with observations. We matched these to U.S. census demographic data for varied spatial scales (state, county, county subdivision) and derived population-weighted aerosol exposure estimates. Aerosol exposure disparities for each demographic and socioeconomic (SES) indicator, with a focus on race-ethnicity and income, were quantified for the period 2013–2020.

**Results:**

The average NYS resident was exposed to 4451 #⋅cm^−3^ UFP and 7.87 μg⋅m^−3^ PM_2.5_ in 2013–2020, butminority race-ethnicity groups were invariably exposed to greater daily aerosol pollution (UFP: +75.0% & PM_2.5_: +16.2%). UFP has increased since 2017 and is temporally and seasonally out-of-phase with PM_2.5_. Race-ethnicity exposure disparities for PM_2.5_ have declined over time; by −6% from 2013 to 2017 and plateaued thereafter despite its decreasing concentrations. In contrast, these disparities have increased (+12.5–13.5%) for UFP. The aerosol pollution exposure disparities were the highest for low-income minorities and were more amplified for UFP than PM_2.5._

**Discussion:**

We identified large disparities in aerosol pollution exposure by urbanization level and socio-demographics in NYS residents. Jurisdictions with higher proportions of race-ethnicity minorities, low-income residents, and greater urbanization were disproportionately exposed to higher concentrations of UFP and PM_2.5_ than other NYS residents. These race-ethnicity exposure disparities were much larger, more disproportionate, and unabating over time for UFP compared to PM_2.5_ across various income strata and levels of urbanicity.

## 1. Introduction

Air pollution is the leading environmental risk factor globally for mortality and disability-adjusted life-years (GBD, 2019). Air pollution is characterized by a mix of toxic gases and aerosols. Atmospheric aerosols or particulate matter are suspensions of tiny particles of solid, liquid, or mixed states with varied sources, compositions, and size distributions. In the last decade, the largest increase in risk exposure has been for ambient particulate matter (PM) pollution (GBD, 2019). The mass concentrations of PM with aerodynamic diameter ≤2.5 μm or PM_2.5_ as an indicator of fine particles, with a health-relevant size range, has been recognized as the most important for air pollution health effects (Fuller et al., 2022; Southerland et al., 2022). However, there is recent increasing evidence that its component ultrafine particles (≤0.1 μm) can have an outsized effect (HEI, 2013; e Oliveira et al., 2019; Moreno-Ríos et al., 2022) despite their small contribution to the total aerosol mass (Seinfeld and Pandis, 2016). These outsized effects that can make ultrafine particles potentially more important than PM_2.5_ are due to their smaller size, higher numbers, larger surface area to volume ratio, higher lung penetration and deposition efficiency, longer atmospheric residence times (when the PM_2.5_ condensation sink is low), and secondary formation from pollutant gases such as acidic sulfur dioxide and nitrogen oxides and alkaline ammonia (Lee et al., 2019). While there may be the implicit policy assumption that PM_2.5_ regulation also regulates their ultrafine component, this is countered by our scientific understanding (Seinfeld and Pandis, 2016) and with evidence that PM_2.5_ and ultrafine particles are not correlated (de Jesus et al., 2019). Yet, ultrafine particles are not regulated or designated as a criteria pollutant. Considering that PM_2.5_ declines may not be accompanied by reductions in ultrafine particles, it is important to comprehensively examine their exposures.

Exposure to air pollution and its health impact associations have disproportionate demographic and socioeconomic influences (Hajat et al., 2015; Tessum et al., 2021). Previous studies have found that minority race-ethnicity and low-income subgroups have larger air pollution exposure for PM_2.5_ (and other criteria pollutants including PM_10_, SO_2_, NO_2_, O_3_, and CO) and associated health effects (Liu et al., 2021; Jbaily et al., 2022) and more recent studies (Saha et al., 2022; Elford and Adams, 2021) have examined the ultrafine particle exposure disparities. These North American studies using land-use regression estimates (Saha et al., 2022; Chambliss et al., 2021; Elford and Adams, 2021) and mobile monitoring (Chambliss et al., 2021) find race-ethnicity and demographic disparities in UFP exposure with the potential for inequality patterns differing from other air pollutants. However, there remain knowledge gaps due to limitations of spatial and temporal scopes and the paucity of ultrafine particle measurements potentially introducing sampling biases. It is therefore important to examine the socioeconomic disparities in ultrafine particle exposure on larger temporal and spatial scales.

To fill the knowledge gaps described above, the objective of our research was to compare the difference between and quantify the socioeconomic disparities for ultrafine particle and PM_2.5_ exposure in New York State (NYS). This study focuses on NYS due to the success of national and state environmental policies in reducing PM_2.5_ concentrations (Rattigan et al., 2016), the consequent impact of cleaner air potentially prolonging the atmospheric lifetime of UFP (Seinfeld and Pandis, 2016), and the availability of a high-quality hospital registry (SPARCS, 2023) for future studies seeking to understand aerosol-health associations and their socio-demographic disparities. We hypothesized that ultrafine particle exposure disparities exist and differ from those of PM_2.5_. Specifically, we examined the spatiotemporal variations in aerosol pollutants (ultrafine particles & PM_2.5_) exposure over NYS from 2013 to 2020, their potential modifications by the urbanization level of residence, and for differential influences by race-ethnicity and income levels.

## 2. Methods

Toward testing our hypothesis, we combine the US Census Bureau’s American Community Survey (ACS) demographic data with modeled ambient aerosol concentrations. We specifically examine exposure disparities among seven race-ethnicity groups and by household income across varied spatial scales (state, county, and county subdivision) and National Center for Health Statistics (NCHS) urbanicity levels in NYS during the period 2013–2020 for the number concentrations of particulate matter with diameter ≤0.1 μm (UFP) and mass concentrations of particulate matter with diameter ≤2.5 μm (PM_2.5_).

### 2.1. Aerosol concentrations data

There is a dearth, in terms of spatial distribution and temporal continuity and resolution, of ultrafine particle measurements in NYS. For this reason, UFP was quantified using a publicly available global model of atmospheric chemistry and transport coupled with a state-of-the-science advanced particle microphysics model that has been comprehensively validated with laboratory and global in-situ measurements. The GEOS-Chem model is a global 3-D model of atmospheric composition driven by assimilated meteorological observations from the Goddard Earth Observing System (GEOS) of the NASA Global Modeling Assimilation Office (GMAO). The model has been developed and used by many research groups and contains a number of state-of-the-art modules treating various chemical and aerosol processes (Bey et al., 2001; Martin et al., 2003; Park et al., 2004; Evans and Jacob, 2005; Liao et al., 2007; Fountoukis and Nenes, 2007) with up-to-date key emission inventories (Guenther et al., 2006; Bond et al., 2007).

The Advanced Particle Microphysics (APM) model, incorporated into GEOS-Chem by Yu and Luo (2009), is an advanced multi-type, multi--component, size-resolved microphysics model. 40 sectional bins represent secondary particles covering dry diameters ranging from 0.0012 μm to 12 μm, with high resolution for the particle size range important for growth of nucleated particles. 20 sectional bins represent sea-salt, covering dry diameters from 0.012 to 12 μm, 15 bins represent dust from 0.03 to 50 μm, 15 bins each for tracking primary black carbon (BC) and organic carbon (OC) from 0.03 to 1 μm separately. Sulfate and other secondary species coating on primary particles such as BC, OC, sea-salt, and dust are considered here. The aging of BC and OC that turns the hydrophobic BC and OC hydrophilic is considered based on the quantity of secondary species coated on them. Aerosols in-air and in-cloud are traced separately. Size-resolved particle microphysics (nucleation, coagulation, condensation/evaporation, and dry and wet deposition) important for aerosols is explicitly considered. The formation of new particles is calculated with state-of-the-science nucleation schemes (Yu et al., 2017, 2018, 2020). The kinetic condensation of low volatility secondary organic gas and H_2_SO_4_ gas on nucleated particles is calculated based on a scheme that considers the volatility changes of secondary organic gases (SOG) arising from the oxidation aging (Yu, 2011). The contributions of nitrate, ammonium, and semi-volatile secondary organic aerosols (SOA) to particle growth are considered. Via the coating process caused by coagulation, condensation, and in-cloud oxidation, secondary species can assimilate with primary particles and be transported and scavenged.

GEOS-Chem-APM has been extensively used, improved, and validated with global atmospheric measurements and robustly quantifies aerosol size distributions, aerosol numbers and aerosol mass (e.g., Yu and Luo, 2009; Nair et al., 2021). We quantify UFP and PM_2.5_ over New York State from 2013 to 2020 by running GEOS-Chem-APM in the nested grid configuration with a spatial resolution of 0.25° × 0.3125° and we output hourly data for the near surface layer in which exposure to aerosol pollution occurs.

### 2.2. Demographic data

Socioeconomic and demographic data was obtained from the publicly available 2015–2019 5-year estimates from the American Community Survey (ACS) owing to the multi-year estimates having higher reliability at smaller spatial scales and for smaller subpopulations. Data access was facilitated by the Census Bureau’s Data APIs and the *tidy-census* package (Walker and Herman, 2023) in R (R Core Team, 2023). Considering the increasing margin of error for population estimates of subgroups with increasing spatial resolution (Wong and Sun, 2013; Spielman and Singleton, 2015), the spatial resolution of the modeled aerosol concentrations, and the eight-year period of study, the most balanced finest spatial scale identified and used in this study is the county subdivision. The mean spatial span of an NYS county subdivision is 0.14° (IQR: 0.10°–0.16°) compared to 0.28° for the GEOS-Chem-APM grid. The variables of interest are the spatial boundaries of geographic areas (state, county, and county subdivision), population estimates by race-ethnicity groups, and median household income. The seven race-ethnicity groups considered here are Hispanic (Hispanics of any race), Asian (non-Hispanic Asian alone), Black (non-Hispanic Black or African American alone), Native (non-Hispanic American Indian or Native American alone), Other (non-Hispanic Other alone or two/more races), Pacific (non-Hispanic Native Hawaiian or other Pacific Islander alone), and White (non-Hispanic White alone). Median household income is used as an indicator of economic status. In this paper, the term race-ethnicity minority refers to the subset of the population that is not White (as defined above). In the analysis presented here, the economic status classifications are Low (<25th percentile), Middle (25th–75th percentile), and High (>75th percentile) median household income, which we derive for each of the above race-ethnicity groups. Supplementary analyses (Text S1) use additional indicators of economic status such as poverty level, house ownership, ratio of income to poverty level, and income brackets, as well as age. Since the link between urbanicity and health outcomes is well-established, the National Center for Health Statistics’ (NCHS) six-level urban-rural classification scheme—four metropolitan (large central, large fringe, medium, and small metro) and two non-metropolitan (micropolitan and noncore)—is applied in the present study.

### 2.3. Study design

#### 2.3.1. Aerosol exposure definition

Demographic and aerosol concentration data were matched by county subdivision boundaries. Aggregation to higher groups (spatial/urbanicity/race-ethnicity/income) was carried out by the population-weighted geometric means, considering the lognormal nature of both UFP and PM_2.5_ distributions and to better capture their central tendency in further aggregations. For example, average exposure was determined using the following formula:

$$E_{ij}=\frac{\sum_{j=1}^{n}\log_{10}\left(C_{j}\right)pop_{ij}}{\sum_{j=1}^{n}pop_{ij}}$$

where, the subscript i indicates the subgroup of interest, the subscript j indicates the jurisdiction (out of n), C is the atmospheric pollutant concentration, and pop is the population. Sensitivity analyses for the temporal scale (Text S2) are additionally carried out with the >90th percentile hourly exposures for each day, since there currently exists no prescribed threshold criteria for UFP, to provide further corroboration of findings.

#### 2.3.2. Disparity metrics definition

All disparity metrics are calculated using the above defined population-weighted aerosol exposure and presented relative to an indicated reference group, which may be the average population or the least exposed sub-group (in this study, Non-Hispanic White). Three metrics are used in-text to quantify disparity: (1) absolute difference in exposures $\left(\Delta E_{ij}=E_{ij}-E_{rj}\right)$, (2) relative percentage difference in exposures $\left(\%E=\Delta E_{ij}/E_{rj}\times 100\%\right)$, and (3) representation bias in demographic proportion as:

$$\text{Representation}\;\text{Bias}=\left(\sum_{j^{90}}\frac{pop_{ij}}{pop_{j}}-\sum_{j=1}^{n}\frac{pop_{ij}}{pop_{j}}\right)\times 100\%$$

where the subscript *i* indicates the subgroup of interest, the subscript r indicates the reference subgroup, the subscript j indicates the jurisdiction, and pop is the population. Here, the representation bias is defined as the percentage difference between a subgroup’s proportion in jurisdictions with >90th percentile pollutant exposure $j^{90}$ compared to all jurisdictions. The third metric is useful to identify if a subgroup (say, a racial minority) has different representation than expected. The three disparity metrics are presented for the urbanicity levels, race-ethnicity groups, income groups or their nested sub-groups as defined in Section 2.2.

#### 2.3.3. Temporal variability

The daily (geometric mean and 90th percentile) aerosol pollutant (UFP & PM_2.5_) concentrations are calculated from the hourly values quantified using GEOS-Chem-APM (Section 2.1.). These are then matched to the population (Section 2.3.1.) and exposure disparities calculated (Section 2.3.2.). To smooth out short-term variabilities arising from the intra-annual effects of meteorology and emissions variability, we present the yearly-moving average, which is the average of daily values (of exposure or its disparity metrics) in a 1-year moving window.

## 3. Results

### 3.1. Spatiotemporal aerosol exposure in NYS, 2013–2020

**Fig. 1.**
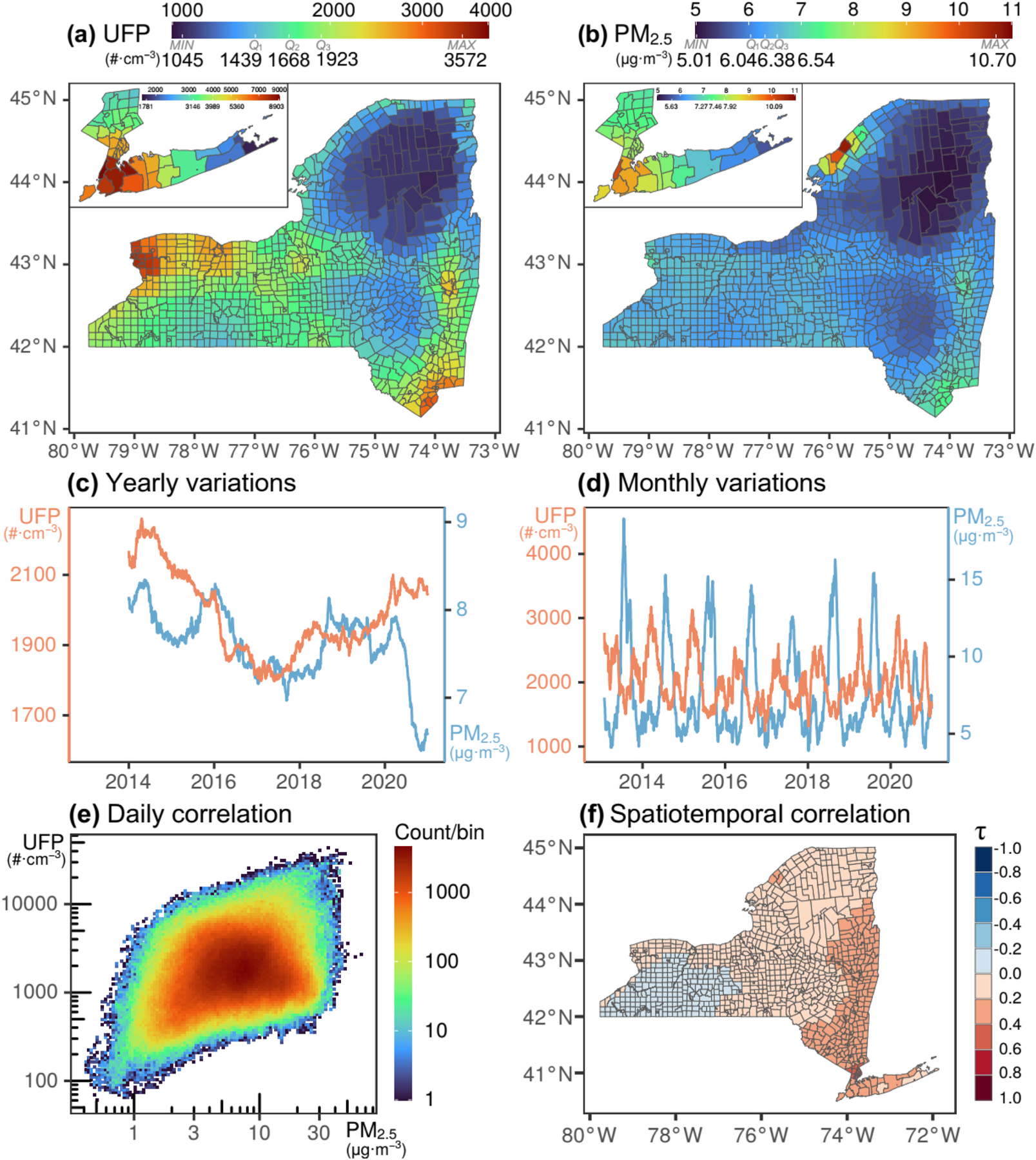
Characteristics of ultrafine particle number concentrations (UFP) and PM_2.5_ over New York State (NYS) from 2013 to 2020. (Top) Spatial distributions of period averaged values for (a) UFP and (b) PM_2.5_. Insets for New York Metropolitan Areas (NYMA). (Center) Time series (moving average) for UFP (orange) and PM_2.5_ (blue) showing (c) yearly and (d) monthly variations of aerosol concentrations over NYS. (Bottom) Extent of daily UFP–PM_2.5_ correlation: (e) binned scatter plot and (f) spatial Kendall rank correlation coefficient (τ) at the county subdivision level.

Fig. 1(a&b) shows the period-averaged (geometric mean) aerosol concentrations (UFP and PM_2.5_) and their large variability over NYS by county subdivision during the period 2013–2020. For NYS overall, their population-weighted values are 4451 #⋅cm^−3^ for UFP and 7.87 μg m^−3^ for PM_2.5_ and their area-weighted values are 1649 #⋅cm^−3^ for UFP and 6.19 μg m^−3^ for PM_2.5_. The largest values are typically over the New York Metropolitan Areas (NYMA) as shown in the insets in Fig. 1(a&b). Table 1 shows that both UFP and PM_2.5_, unsurprisingly, increase with the level of urbanization. The yearly-moving average, which smooths out short-term variabilities (including the intra-annual effects of meteorology and emissions variability), is shown in Fig. 1(c). The monthly moving average, in Fig. 1(d), shows that UFP and PM_2.5_ are also seasonally out of phase. While UFP and PM_2.5_ may have appeared to be correlated in Fig. 1(a&b), when considering the temporal dimension (daily), there is no/weak correlation as seen in Fig. 1(e). This translates to no/weak spatiotemporal association between UFP and PM_2.5_ as illustrated in Fig. 1(f). Even for longer temporal scales, such associations are weak (Fig. 1(c&d)). These serve to illustrate the dissimilar trends and variations of UFP and PM_2.5_ and additionally uncover that their reductions have tapered out in recent years.

### 3.2. Aerosol exposure disparities by race-ethnicity and urbanicity

**Fig. 2.**
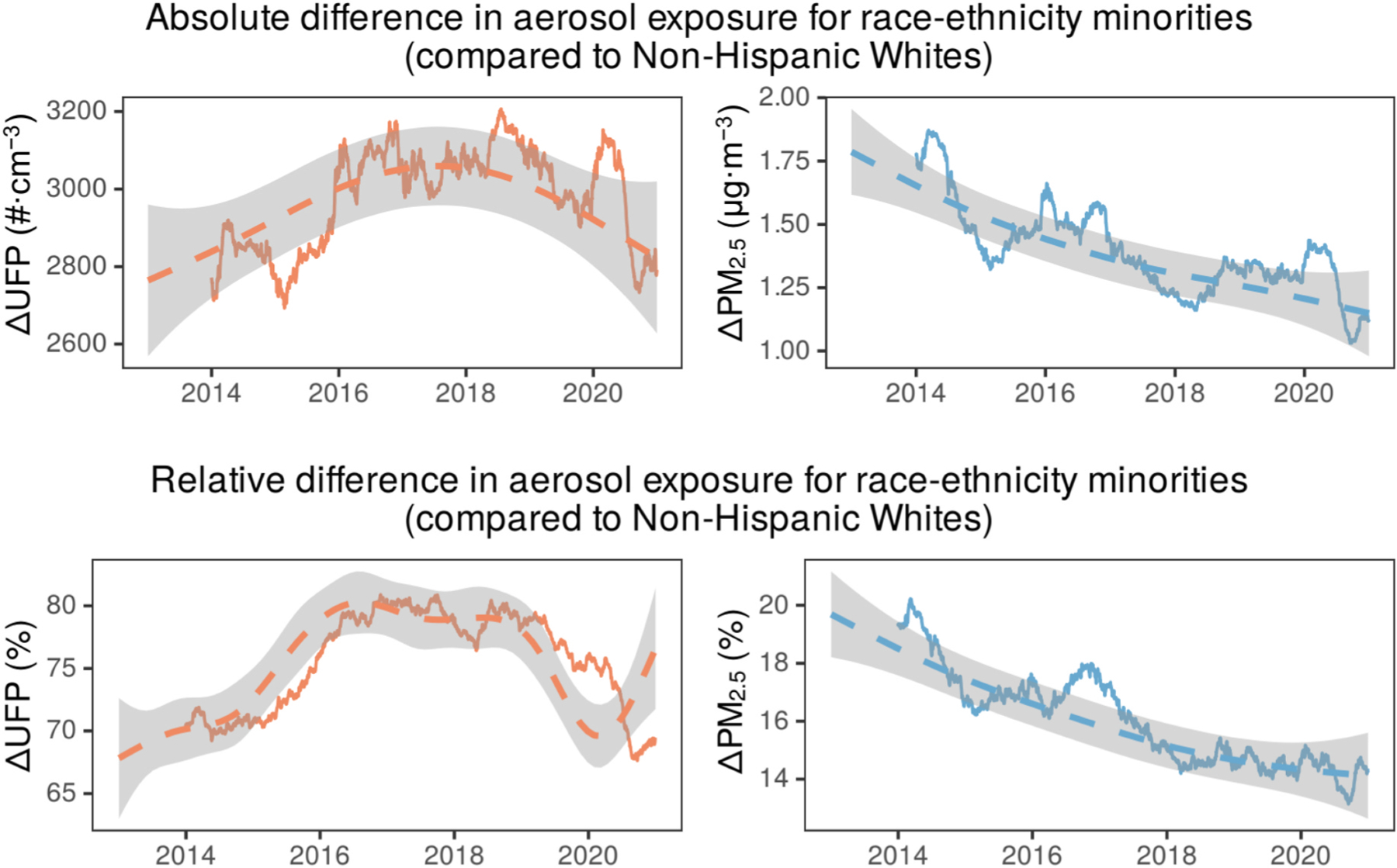
Yearly moving average for the temporal evolution of race-ethnicity disparities in aerosol pollutant exposure in (top) absolute terms and (bottom) relative terms. Dashed curve indicates the generalized additive model (GAM) fits on the daily data and the associated shading in grey the 95% C.I. for the fits. Disparities are presented for the aggregated race-ethnicity minority group compared to non-Hispanic White subgroup. Socio-demographic information is from the American Community Survey 2015–2019 5-year data. Shown on the left (orange) are these for UFP and on the right (blue) for PM_2.5_. UFP exposure disparities are larger and unabating as compared to those for PM_2.5_.

Fig. 2 shows the aerosol exposure disparities for aggregated race-ethnicity minorities compared to the non-Hispanic White subgroup. UFP exposure disparities are large in absolute (2600–3200 #⋅cm^−3^) and relative (65–80%) terms. In comparison, PM_2.5_ exposure disparities during this period are 1–2 μg m^−3^ in absolute terms and 14–20% in relative terms. Apart from the observed larger magnitude of UFP exposure disparities (in both absolute and relative terms) compared to PM_2.5_, there are differences in their temporal trends illustrated in Fig. 2 using generalized additive model fits. PM_2.5_ exposure race-ethnicity disparities (absolute & relative) show a continuously declining temporal trend. For UFP, however, there is no statistically significant trend in the absolute disparity and indicating that in addition to the larger magnitude they remain unabating over time. In relative terms, the UFP disparities are increasing from 2013 to 2017, plateauing at their high values from 2017 to 2019, declining in 2019, and again increasing in response to the concentration reductions during the COVID-19 pandemic period. While illustrative of the overall differences, we will not dwell further on these observations, since the effect of urbanization level on health outcomes and pollutant exposure are important, and it is crucial to also consider urbanicity when exploring these disparities.

**Fig. 3.**
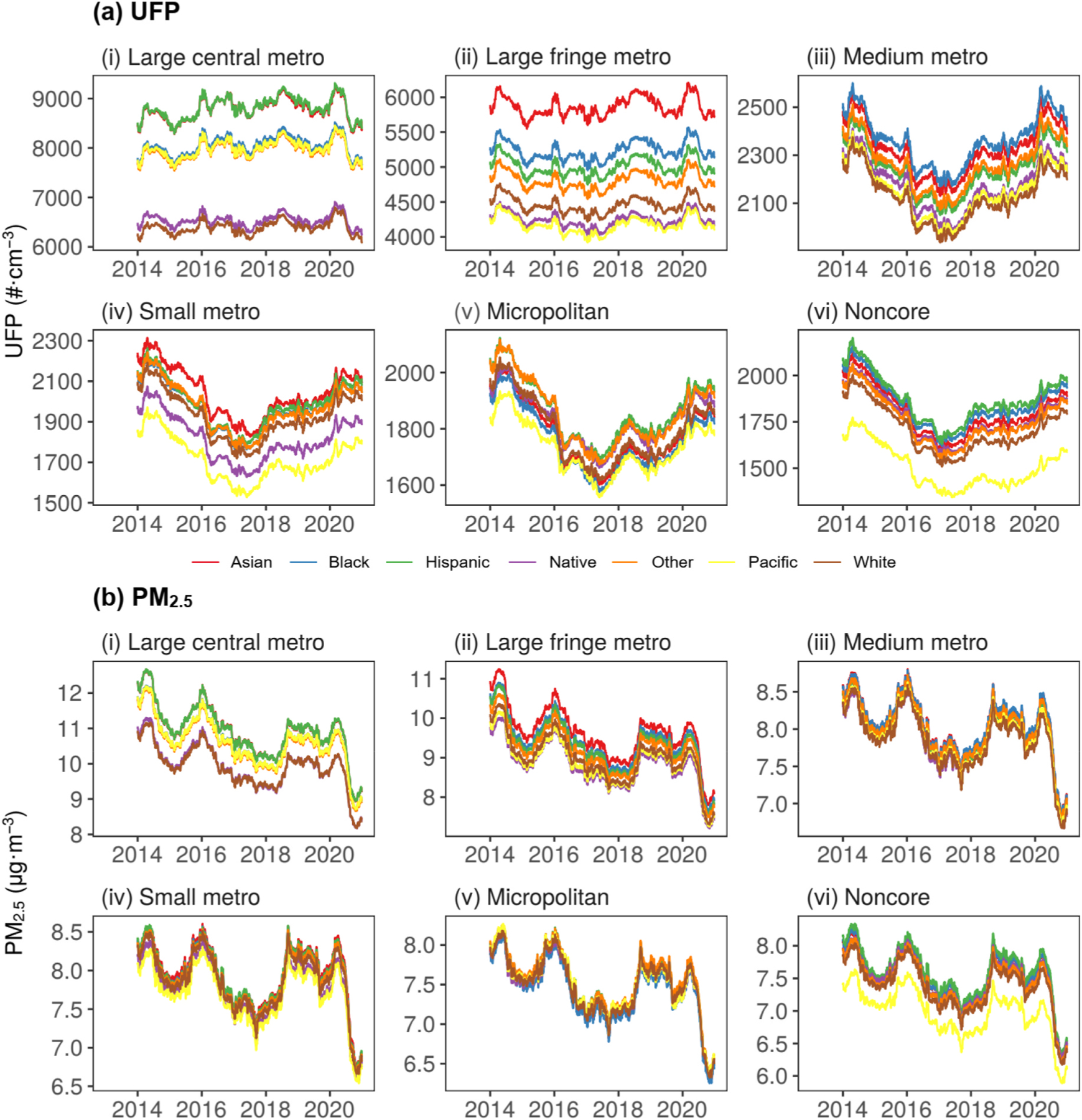
Yearly moving average for (a) UFP and (b) PM_2.5_ population-weighted exposure at the county subdivision level in NYS during 2013–2020. Shown for each race-ethnicity group (color legend) with facets corresponding to each NCHS urbanization level (i–vi).

Fig. 3 shows the trend and absolute magnitude of population-weighted aerosol exposure over NYS for each race-ethnicity group (see Table 1) with facets corresponding to the NCHS urbanicity levels. In large (central and fringe) metropolitan areas, UFP exposure has been the highest and remained mostly constant over the eight-year period (Fig. 3 (a)-i–ii). Linear fits on Fig. 3 (see Supplementary Fig. S1) to estimate the temporal percent change (Table 2) show small increases (2013–2020: 0.0 to +2.2%; 2013–2019: −0.8 to +3.1%) for minority race-ethnicity subgroups and a smaller increase (2013–2020: +0.69%) for the White subgroup. This is unlike that for PM_2.5_ (Fig. 3(b)-i–ii), which has demonstrated large decreases over the period for minority race-ethnicity (2013–2020:−14.7 to −13.1%; 2013–2019: −9.9 to −11.9%) and White (2013–2020: −12.8%; 2013–2019: −9.6%) subgroups. In areas of lower levels of urbanization (Fig. 3(a)-iii–vi), the initial decline (−17.7–20.4%) in UFP starts a reversal from the year 2017 onwards with increasing (2017–2020: +12.5 to +13.5%; 2017–2019: +6.0 to +7.4%) absolute UFP exposure for all race-ethnicity subgroups. For PM_2.5_ (Fig. 3(a)-iii–vi), ignoring the reduction in 2020 associated with the COVID-19 pandemic period reduction of its (and precursor) emissions, the initial decline (2013–2017: −8.5 to −7.7%) is followed by a reversal (+7.5 to +8.2%). Invariably, the average NYS resident belonging to the Black, Hispanic, Asian, and Other race-ethnicity groups are exposed to higher absolute UFP than White individuals (Fig. 3(a)). In large (central and fringe) metropolitan areas, which are areas of high UFP concentrations, these exposure disparities are largest, particularly for Asians (Fig. 3(a)-i–ii). Although these exposure inequalities are also demonstrated for PM_2.5_, they are not as large as those for UFP.

**Fig. 4.**
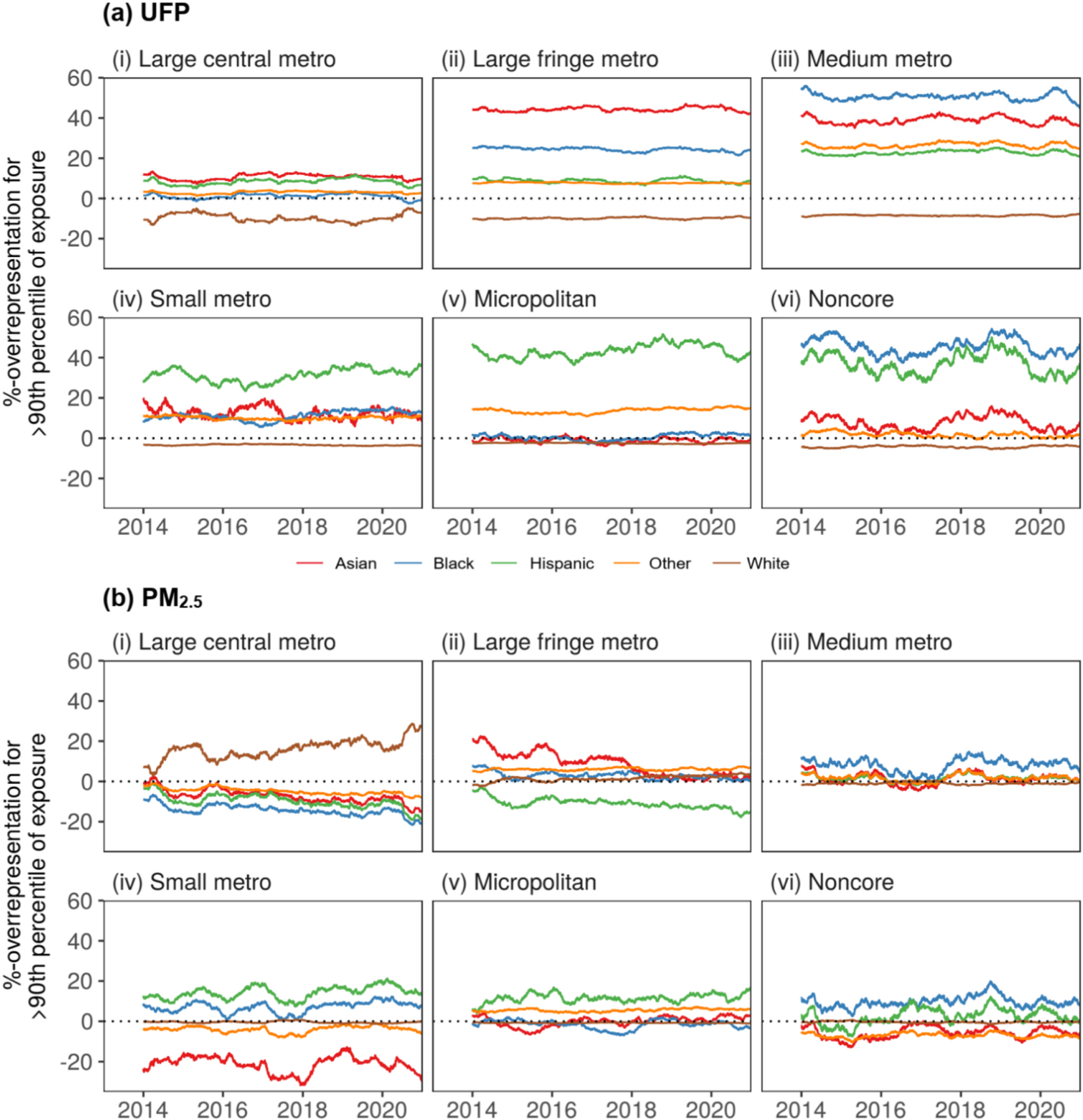
For (a) UFP and (b) PM_2.5_ population-weighted exposure at the county subdivision level in NYS during 2013–2020, the representation bias for each race-ethnicity group (color legend) in the worst 10% exposure jurisdictions corresponding to each NCHS urbanization level (i–vi). Values for Native and Pacific groups are omitted due to small sample sizes resulting in high variability.

Fig. 4 shows the race-ethnicity overrepresentation percentages for the worst-aerosol-exposed jurisdictions for each NCHS urbanicity level. Since UFP is not a designated criteria pollutant and the limited number of existing epidemiological studies show wide variability in discerning a critical threshold, we use an arbitrary 90th percentile cutoff for daily highest UFP exposure for county subdivisions grouped at the NCHS urbanicity level. Fig. 4(a) shows that across all levels of urbanization, the non-Hispanic White group is underrepresented in the jurisdictions with the top 10% UFP exposure. Typically, these high-exposure county subdivisions are populated by more minority race-ethnicity groups than average. A similar analysis is presented in Fig. 4(b) for PM_2.5_, to place the findings for UFP illustrated in Fig. 4(a) in context. Fig. 4(b) demonstrates that the disparities in PM_2.5_ exposure do not reflect those in UFP exposure. PM_2.5_ exposure disparities by race-ethnicity have been declining in absolute and relative terms. Furthermore, these disparities are negligible outside large metropolitan areas. Classification by the NCHS urban levels and not simply by a binomial factor of urban/rural also reveals that in metropolitan areas, with some of the highest (>90th percentile) PM_2.5_ burden, the relative exposure disparities for minority race-ethnicity groups (Fig. 4(b)-i–ii) are lower and furthermore have been reducing over time. This contrasts with the observation for UFP exposure disparities (Fig. 4(a)-i–ii), which are higher and have remained fairly unchanged or slightly increasing over the period.

### 3.3 Large race-ethnicity disparities in aerosol exposure are magnified in low-income groups

**Fig. 5.**
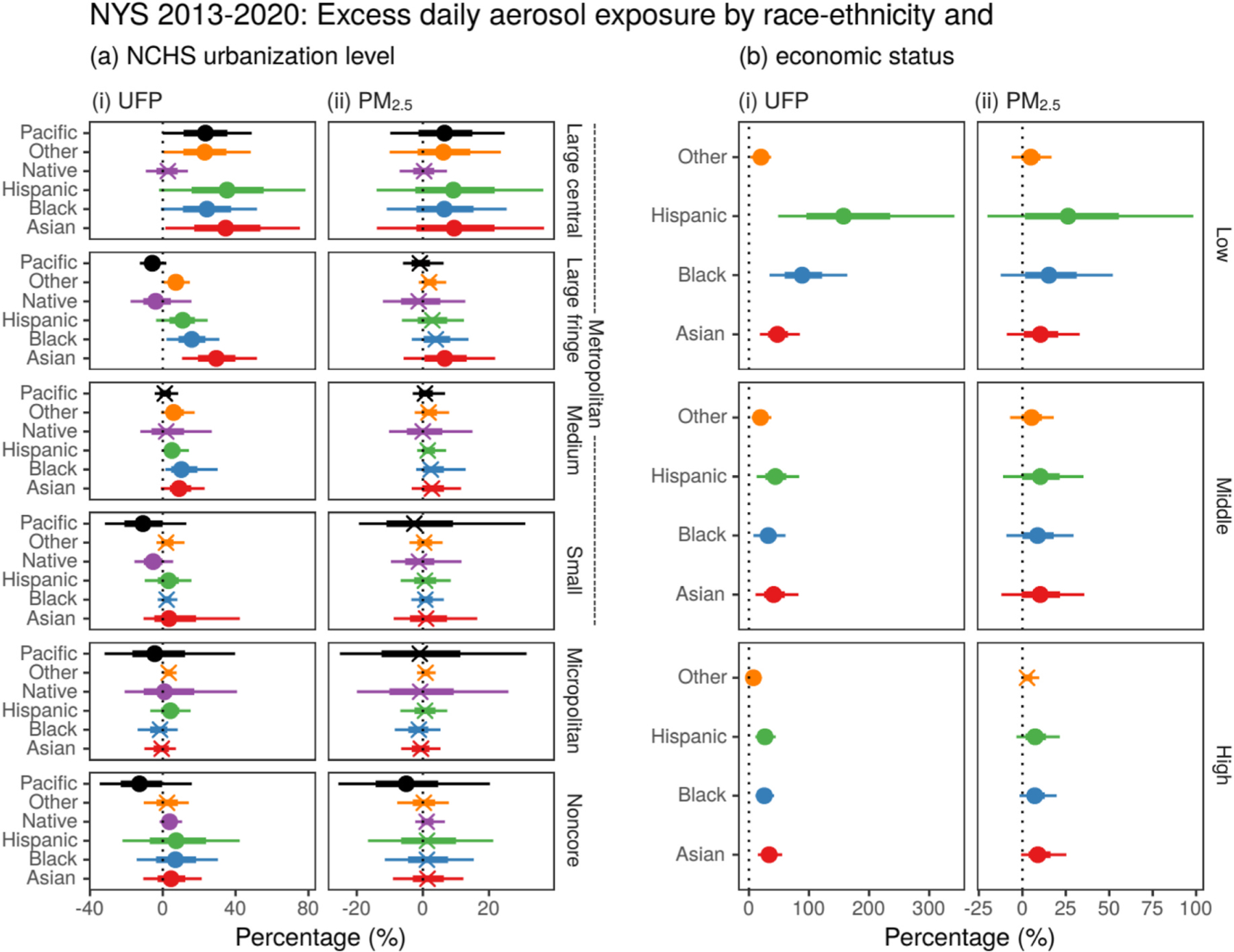
The relative excess exposure (%) to aerosol pollution for race-ethnicity groups compared to the non-Hispanic White subgroup by (a) urbanicity and (b) economic status at the county subdivision level in New York State. Circles indicate a statistically significant difference between distributions (each vs. non-Hispanic White) and crosses otherwise.

Fig. 5(a) illustrates the relative excess daily aerosol exposure for an average minority race-ethnicity individual compared to an average non-Hispanic White individual for each urbanization level. The disparity is much greater for UFP (Fig. 5(a)-i) than for PM_2.5_ (Fig. 5(a)-ii). The only overall significant PM_2.5_ race-ethnicity exposure disparities are for the large central metro jurisdictions. For UFP, these disparities are persistent and increase as the level of urbanization increases. Fig. 5(b) compares these excess daily aerosol exposures for minority race-ethnicity individuals for each economic status classifications relative to non-Hispanic White individuals in the corresponding classification, i.e., Low-income Hispanic to Low-income non-Hispanic White and so on. This further illustrates that the race-ethnicity disparities persist across income levels and are dramatically magnified in economically vulnerable groups (lowest quartile) for aerosol pollution and are greatest for UFP.

## 4. Discussion

### 4.1. Characterizing UFP and need to understand its exposures

In this study, we characterize ultrafine particles by number concentration (UFP) rather than mass concentration (PM_0.1_). Ultrafine particles, by virtue of their small sizes, may not carry much mass. For instance, ~1.6 × 10^4^ particles of 0.1 μm carry as much mass as a single particle of 2.5 μm. From microphysical considerations, although the aerosol mass contribution of ultrafine particles may be small, they contribute to the largest number of ambient atmospheric particles. Additionally, ultrafine particles, especially those between 0.05 and 0.1 μm, can dominate the net particle surface area. Aside from the microphysics, the changing atmospheric environment, that of its declining condensational sinks, increasing ambient atmospheric alkaline ammonia (Nair and Yu, 2020) and tapering reductions in the acidic sulfur dioxide and nitrogen oxide gases (for secondary particle formation and growth) and other factors such as criteria air pollutant mitigation strategies (Cheng et al., 2019) that may inadvertently induce new particle formation, may further increase the preeminence and role of UFP. Aside from the microphysics and the changing atmospheric environment, in-vitro and modeling studies demonstrate that UFP, largely due to their unique microphysical properties, are efficient in penetrating the respiratory tract (Peters et al., 2006; Sturm, 2016a; Traboulsi et al., 2017), capable of inducing large physiological stress and inflammation (Leikauf et al., 2020; Schraufnagel, 2020), and demonstrate higher ability to cross barriers (Terzano et al., 2010; Chen et al., 2016; Selvaraj et al., 2018; Bongaerts et al., 2020) such as the alveolar-blood, blood-brain, nose-to-brain pathways, placental membranes, and even down to the subcellular level, into cells and its organelles (Ohlwein et al., 2019 and the references therein). Furthermore, additional microphysical considerations such as probability and location of deposition in the respiratory tract (Darquenne, 2012; Sturm, 2016a, 2016b), particle-membrane interactions (Oberdörster et al., 2005) such as localized exposure, adsorption, and diffusion deems it appropriate to characterize ultrafine particles by their number concentration as UFP. To digress, epidemiological studies exploring the effects of atmospheric aerosols initially observed that PM_2.5_ was a more robust parameter than PM_10_ for assessing health impacts; this was later understood to be due to its size-related microphysical properties. It may therefore be pertinent that our efforts at understanding the relationships between aerosols and health effects place weight on the aerosol size-dependencies, especially on the more numerous smaller-sized aerosols.

### 4.2. UFP and PM_2.5_ exposure and their differences

UFP and PM_2.5_ show no/negligible spatiotemporal correlation in New York State. In fact, the two are seasonally out of phase, with UFP being more dominant in the colder months. We also found that UFP has gradually increased since 2017 even during periods of PM_2.5_ decline in recent years. Pollution mitigation policies were expected to simultaneously reduce direct emissions of both PM_2.5_ and UFP as well as emissions of their precursor gases (for secondary aerosol formation). The observed increase in UFP is, however, in agreement with our understanding (Seinfeld and Pandis, 2016) that with the reduction of larger particles (~PM_2.5_) acting as coagulation sinks for smaller particles (~UFP), their atmospheric lifetime thus increases, as well as with observations (Chen et al., 2022). There may be additional inadvertent impacts of pollution control conducive to secondary UFP formation from cleaner gasoline (Zhao et al., 2017) and ammonia from Compressed Natural Gas (CNG) combustion (Nair and Yu, 2020), with the latter also having higher (than diesel) potential for direct emissions of UFP (Jayaratne et al., 2008) vehicle exhaust. By demonstrating the differential spatiotemporal variabilities, we highlight the potential fallacy in not designating UFP as criteria pollutant, possibly under the assumption that controls for PM_2.5_ would mitigate UFP. Adding the fact that the leading causes of deaths and morbidity are of the cardiovascular and cerebrovascular nature (e.g., GBD, 2019; Lin et al., 2022), and that these have been identified to be elevated in colder months (e.g., Lin et al., 2018; Alahmad et al., 2023), UFP may have further outsized health effects, especially for New York State.

### 4.3. Aerosol exposure disparities by urbanicity

We uncover that the aerosol exposure inequalities by the urbanization level of residence are more amplified for UFP exposure compared to that for PM_2.5_. While there are varied methods to categorize urbanicity, we use the 2013 NCHS urban-rural classification scheme. This classification is robust as it considers the nuanced urban-rural differences in health measures. Unsurprisingly (Brender et al., 2011; Bell and Ebisu, 2012; Colmer et al., 2020), due to the strong influence of anthropogenic sources of aerosols and their precursors, as the level of urbanization increases, so does the pollutant aerosol exposure. However, the extent of the urban-rural divide in aerosol exposures is found to be much larger and unabating for UFP than that for PM_2.5_.

### 4.4. Aerosol exposure disparities by urbanicity and race-ethnicity or economic status

Overall, for NYS, non-Hispanic Whites were least exposed to UFP and PM_2.5_. Non-Hispanic Asian, Hispanics of any race, and Non-Hispanic Black or African American subgroups have especially disparate exposures, with median exposures higher by 88%, 77%, and 70% for UFP and 15%, 14%, and 13% for PM_2.5_, respectively. The large magnitudes of UFP exposure disparities uncovered here are important considering the future projected decreases of PM_2.5_ and increase of UFP (Turnock et al., 2020) and the excess risks of deleterious health outcomes associated with UFP (Ohlwein et al., 2019). Typically, PM_2.5_ exposure disparities by race-ethnicity have been declining in absolute and relative terms in agreement with previous studies (Liu et al., 2021; Jbaily et al., 2022). Specifically for NYS, Liu et al., 2021 find a −1.33 μg m^−3^ decline in the absolute PM_2.5_ disparity from 2000 to 2010 and 10–20% higher PM_2.5_ exposure for minorities. However, we also find that in recent years (2017–2019), there has been a reversal of trend for the absolute PM_2.5_ exposure disparities but in relative terms the decline has continued albeit plateauing. It is important, however, to also examine these disparities by urbanicity due to differences in absolute exposure levels and studies showing varied health outcomes. Since race-ethnicity minorities and low-income populations predominantly reside in urban areas, analysis without stratification by urbanization level may inadvertently inflate the aerosol exposure disparity estimates. Examination of race-ethnicity exposures separately for each NCHS urbanicity level, reveals that these minority subgroups are consistently the most exposed to UFP and PM_2.5_ across all levels of urbanicity in absolute and relative (to the least exposed subgroup) terms. The findings are corroborated with a sensitivity analysis using the 90th percentile (in the absence of a UFP threshold) of daily exposures identifying the most-exposed jurisdictions, which have a larger minority race-ethnicity proportion than on average. We find that economic status indicators of household income, poverty level, ratio of income to poverty level, and homeownership (after accounting for urbanicity differences) by themselves are not associated with significantly different UFP exposures.

### 4.5. Aerosol exposure disparities by urbanicity, race-ethnicity, and income

Under the additional lens of economic status, we see that these effects are magnified for minority race-ethnicity subgroups within the lowest quartile of household income relative to non-Hispanic Whites. This is consistent with previous studies examining the impact of income inequalities on PM_2.5_ (Liu et al., 2021; Jbaily et al., 2022) and UFP (Saha et al., 2022). We find that income inequalities alone are not associated with significantly different UFP exposures, unless in interaction with race-ethnicity. Although exposure disparities have reduced over time for PM_2.5_, they have been increasing for UFP, and especially so in recent years. This may translate to more adverse aerosol-health effects for race-ethnicity minorities and vulnerable socioeconomic subgroups in an atmosphere deemed seemingly cleaner by air quality regulations. Indeed, there is mounting recent evidence that the benefits from clean air regulations are not translated proportionately to reductions in the associated health burden for minority race-ethnicity and SES subgroups. These findings may be linked to the atmospheric aerosol size distributions skewing toward smaller sizes with outsized health effects and requires our immediate and further attention.

Overall, we find that the aerosol exposure disparities were larger and more disproportionate for UFP compared to PM_2.5_. This observation arises from the differences in their spatial distribution. PM_2.5_ is typically regionally homogenous, with its composition being predominantly secondary after undergoing growth and/or aging processes (Fine et al., 2008; Kim et al., 2020). While UFP is driven by regional new particle formation (nucleation) events, their concentrations are also impacted by local sources (particularly roadways) of primary (directly emitted) UFP as well as its gaseous precursors. Thus, there exists a greater degree of spatial heterogeneity in UFP, particularly important over urban areas (Gomišček et al., 2004; Puustinen et al., 2007; Wu et al., 2015; de Jesus et al., 2019). While local interventions can reduce concentration extremes experienced by race-ethnicity minorities, reduction of background UFP can be more effective at addressing population-wide race-ethnicity exposure disparities (Chambliss et al., 2021).

This study has the following key strengths. One, this is the first study identifying and quantifying the spatiotemporal socio-demographic disparities in ultrafine particle exposure. Two, a state-of-the-science model of atmospheric chemistry and transport is used to quantify atmospheric aerosol concentrations. An advantage of this model is the physical and chemical consistency in the quantification of pollutant aerosols and copollutant gases that may be lacking even in sets of direct measurements, due to variability in instrumentation protocols and other sources of error. Three, the large spatial scale, that of New York State, permits sensitivity analyses with respect to different spatial resolutions: the county subdivision and the coarser county level. Across these scales, the findings remain consistent. Four, the high temporal resolution permits sensitivity analyses that allows for exploration of disparities for high aerosol exposure.

This study has the following limitations. One is the spatial scale; here, the finest spatial resolution is the county subdivision level. This choice is for two reasons: (1) for spatial levels smaller than the county subdivision, demographic estimates are subject to large margins of error due to the lower population of subgroups and (2) the model simulated aerosol concentrations presently do not resolve the tract level exposures. This coarser resolution may smooth out true exposures and their disparities at smaller scales, all the way to the individual-level. For UFP, however, with the impact of regional nucleation events and the longer overall (when the PM_2.5_ condensation sink is low) atmospheric lifetime, its concentration is expected to be smoothed out over several kilometers. In areas with local UFP sources, such as near roadways and airports, the coarser resolution may result in underestimation of the exposure. Clark et al. (2022) found that national air pollution exposure disparity estimates based on state and county scale data could substantially under-estimate those estimated using tract-level or finer scales. The disparities calculated in this study may therefore be underestimates. Also, in using the five-year ACS estimates for socio-demographic variables, we cannot account for changes in the population at the county subdivision level occurring during the period of the study. Two, owing to the dearth of comprehensive long-term in situ measurements at present, we currently have to rely on physically and chemically consistent models of atmospheric chemistry and transport that have been extensively validated with laboratory and available in situ observations for studying aerosol exposure impacts on non-localized scales from global to national to regional scales. The current dearth of UFP measurements also means that there remains large scope for improvement of land-use regression (LUR) and other spatiotemporal exposure models, satellite inferences, and hybrid modeling for UFP exposure assessment. There is hope, however, for improved exposure assessment and consequent health impacts in the coming years through the newly established long-term, ground-based high time-resolution ASCENT (Atmospheric Science and Chemistry mEasurement NeTwork) air quality monitoring network, which will start to address these knowledge gaps in the US. Three, the exposure disparities estimated here connect to residency and do not include mobility and other sources of personal exposure (such as smoking, indoor appliances, or air filtration systems) that unfortunately are not available. There may be confounding factors resulting in a difference between aerosols outdoor and indoors, where an individual spends most of their time on average. Underlying psycho-socioeconomic reasons for the identified disparities may also be important when connecting exposure disparities to health disparities.

## 5. Conclusions

We found that in New York State during 2013–2020, UFP and PM_2.5_ show no spatiotemporal correlation, with PM_2.5_ typically declining but UFP increasing since 2017 and being seasonally out-of-phase with PM_2.5_. This study uncovered that the race-ethnicity disparities for ultrafine particle exposure are large and persistent, across urbanization levels, and that these disparities have increased and even widened during periods of UFP reductions. This is unlike that for fine particles (PM_2.5_), disparities for which have declined over time and plateaued thereafter despite decreasing PM_2.5_ concentrations. Invariably, a minority race-ethnicity and/or low-income group is exposed to the largest aerosol exposure disparity. In the 10% worst jurisdictions in terms of aerosol exposure, reside larger proportions of race-ethnicity minorities than expected. Income disparities magnify the race-ethnicity disparities uncovered in aerosol exposure. These aerosol exposure disparities by race-ethnicity were much larger, more disproportionate, and unabating over time for UFP compared to PM_2.5_ across various income strata and levels of urbanicity.

## Supplementary Material

Environmental disparities

Appendix A. Supplementary data

Supplementary data to this article can be found online at. https://doi.org/10.1016/j.envres.2023.117246.

## Figures and Tables

**Table 1:** Distribution statistics for daily population-weighted UFP and PM_2.5_ exposure in New York State during 2013–2020 by urbanicity and race-ethnicity groupings (for other socioeconomic factors see Table S1). Total population for NYS is 19,572,319 and the percentage of population (%-pop.) is rounded to two decimal places. UFP is rounded to a whole number and PM_2.5_ is rounded to two decimal places.

		% pop.	UFP (#⋅cm^−3^)					PM_2.5_ (μg⋅m^−3^)				
			Exposure Percentiles									
			10th	25th	50th	75th	90th	10th	25th	50th	75th	90th
**Urbanicity**												
Metropolitan	Large central	51.50	3558	4875	6781	9573	12508	4.00	5.81	8.92	13.51	18.96
	Large fringe	27.83	2111	2945	4199	5886	8035	3.24	4.78	7.62	11.79	17.24
	Medium	9.33	958	1383	1957	2761	3711	2.82	4.33	6.77	10.06	14.39
	Small	5.02	916	1208	1623	2248	3002	2.83	4.17	6.48	9.46	13.60
Micropolitan		1.99	791	1119	1544	2171	2988	2.68	4.00	6.29	9.34	13.47
Noncore		4.33	868	1251	1745	2462	3350	2.79	4.23	6.74	10.07	14.52
**Race-ethnicity**												
Hispanic		19.01	3195	4439	6195	8805	12001	3.83	5.57	8.69	13.23	18.76
Non-Hispanic	Asian	8.35	3390	4722	6588	9377	12580	3.95	5.69	8.83	13.45	19.10
	Black	14.26	3110	4337	5962	8469	11228	3.82	5.57	8.63	13.05	18.41
	Native	0.24	1991	2592	3498	4736	6165	3.40	4.90	7.64	11.12	15.74
	Other	2.51	2547	3467	4733	6668	8729	3.64	5.28	8.19	12.26	17.27
	Pacific	0.03	2420	3284	4438	6216	8109	3.65	5.24	8.15	12.10	17.18
	White	55.61	1908	2609	3505	4822	6320	3.37	4.86	7.65	11.14	16.16

**Table 2:** Annual change (%) in aerosol pollutant concentrations by race-ethnicity and urbanicity. Urbanicity is dichotomized into Large (combined large central and large fringe metropolitan areas) and Lesser (combined medium and small metropolitan, micropolitan, and non-core areas). Percent change is estimated from the slope of linear fits (see Supplementary Figs. S1 and S2) on data presented in Fig. 3 for the time periods indicated below. Italicized text for percentage change estimated from fits with *p*-value ≥0.05. Boldface text for percentage change estimated from fits with *R* ≥ 0.5.

Race	Urban	ΔUFP (%)					ΔPM_2.5_ (%)				
		Overall Period		2017 inflection			Overall Period		2017 inflection		
		2013 to 2020	2013 to 2019	2013 to 2017	2017 to 2020	2017 to 2019	2013 to 2020	2013 to 2019	2013 to 2017	2017 to 2020	2017 to 2019
*Supplementary Fig.:*		S1(a)	S2(a)	S1(b)	S1(c)	S2(c)	S1(a)	S2(a)	S1(b)	S1(c)	S2(c)
Asian	Large	2.2	3.1	2.6	−2.5	−1.5	−**14.7**	−**11.9**	−**11.5**	−3.6	**7.9**
	Lesser	−2.2	−**8.5**	−**17.8**	**13.2**	**7.4**	−7.5	−4.9	−**8.2**	−2.4	**8.1**
Black	Large	2.2	2.8	1.4	−1.7	−0.7	−**13.9**	−**11.0**	−**11.3**	−3.6	**8.1**
	Lesser	−1.8	−**8.2**	−**17.7**	**13.2**	**7.3**	−7.6	−4.9	−**8.3**	−2.6	**8.0**
Hispanic	Large	2.2	2.7	1.0	−1.8	−0.9	−**14.5**	−**11.7**	−**11.7**	−3.3	**8.3**
	Lesser	−3.0	−**9.5**	−**19.5**	**13.1**	**7.2**	−7.9	−5.0	−**8.1**	−2.8	**8.2**
Native	Large	*0.0*	−0.8	−3.2	*0.4*	*0.1*	−**13.1**	−**9.9**	−**11.3**	−4.0	**8.1**
	Lesser	−4.5	−**10.7**	−**18.3**	**13.0**	**6.9**	−**8.5**	−5.5	−**8.5**	−3.3	**7.5**
Other	Large	1.8	1.9	−*0.1*	−1.2	−0.6	−**13.8**	−**10.8**	−**11.4**	−3.7	**8.1**
	Lesser	−3.1	−**9.6**	−**19.0**	**13.3**	**7.2**	−7.7	−4.9	−**8.2**	−2.7	**8.1**
Pacific	Large	1.5	1.6	−**0.4**	−0.9	−0.4	−**13.5**	−**10.5**	−**11.2**	−3.7	**8.1**
	Lesser	−5.0	−**11.4**	−**19.8**	**12.5**	**6.0**	−**8.1**	−5.2	−**7.7**	−2.9	**7.9**
White	Large	0.7	−*0.2*	−**3.4**	0.7	0.4	−**12.8**	−**9.6**	−**11.0**	−3.9	**8.3**
	Lesser	−5.1	−**11.7**	−**20.4**	**13.5**	**6.9**	−**8.3**	−5.3	−**8.3**	−3.0	**8.0**

## Data Availability

Data will be made available on request.
