# Supplementary material for: Environmental exposure disparities in ultrafine particles and PM2.5 by urbanicity and socio-demographics in New York state, 2013–2020
Source: Environ Res. Author manuscript; Available in PMC 2025 Jun 25. (PMC12191151; doi:10.1016/j.envres.2023.117246)
Supplement: Environmental disparities [file NIHMS2080553-supplement-Environmental_disparities.pdf]

Supplementary Material for

Environmental exposure disparities in ultrafine particles and $PM_{2.5}$ by urbanicity and socio-demographics in New York State, 2013–2020

Arshad Arjunan Nair [a,*], Shao Lin [b,c], Gan Luo [a], Ian Ryan [c], Quan Qi [d], Xinlei Deng [c], Fangqun Yu [a,*]

[a] Atmospheric Sciences Research Center, University at Albany, State University of New York, Albany, New York 12226, USA
[b] Department of Environmental Health Sciences, University at Albany, State University of New York, Rensselaer, New York 12144, USA
[c] Department of Epidemiology and Biostatistics, University at Albany, State University of New York, Rensselaer, New York 12144, USA
[d] Department of Economics, University at Albany, State University of New York, Albany, New York 12222, USA

Contents

- Text S1 and corresponding Table S1 and Figure S1–2
- Text S2 and corresponding Figures S3–7
- Text S3 and corresponding Table S2 and Figures S8–11

* Address correspondence to Arshad Arjunan Nair and Fangqun Yu, Atmospheric Sciences Research Center, 1220 Washington Ave, Albany NY 12226 USA. Telephone: +1 (518) 437-8719 and +1 (518) 437-8767. Email: aanair@albany.edu and fyu@albany.edu.

Supplementary text

Text S1. Supplementing the analysis in the main manuscript are Tables S1 and Figs. S1–2. Table S1 presents the information as in main Table 1 for additional demographic and economic status indicators. Figure S1 illustrates the larger and unabating UFP exposure temporal variations as compared to those for $PM_{2.5}$ as well as the urban–rural divide and differences prior to and after 2017. Figure S2 is the same as Figure S1, except with the aerosol data for 2020 removed to not consider the changes associated with the COVID-19 pandemic.

Text S2. Sensitivity analysis is conducted by replacing the daily average (geometric mean) exposures with the average (geometric mean) of the >90th percentile hourly exposures for each day. This measure is hereafter denoted as UFP^{90} and $PM_{2.5}^{90}$ for UFP and $PM_{2.5}$, respectively. Figure S3, as in main Fig. 2, shows the larger absolute and relative disparities for UFP^{90} exposure in the minority race-ethnicity group as compared to non-Hispanic Whites. These disparities are persistent and increasing over time. Figure S4 is as that for main Fig.3, except for the highest 10% of hourly aerosol exposures each day (UFP^{90} & $PM_{2.5}^{90}$). The inequalities are magnified but retain similar temporal variability and trends, consistent with the results presented in the main manuscript, and affirming them. Figure S5 illustrates the trends in the period of UFP decrease and increase in non-Large-metropolitan regions of New York State as well as its differences with respect to $PM_{2.5}$. Figures S6 and S7, as in main Fig. 5, illustrate the relative excess exposure to aerosol pollutants by combined urbanicity and race-ethnicity groupings and income and race-ethnicity groupings. Significant and larger inequalities are observed for UFP exposure as compared to $PM_{2.5}$.

Text S3. The analysis in the main manuscript is also presented for the coarser resolution of the county-level. Figure S8 is as in main Fig. 1(a–d), Table S2 as in Table S1, Fig. S9 as in main Fig. 2, Fig. S10 as in main Fig. 3, and Fig. S11 as in main Fig. 5(a)

Supplementary tables

Table S1. County-subdivision-wise distribution statistics for daily population-weighted UFP and $PM_{2.5}$ exposure in New York State by age, race-ethnicity, economic status indicators, and urbanicity. Percentage of population (%-pop.) is rounded to two decimals. UFP is rounded to whole number and $PM_{2.5}$ is rounded to two decimal places.

			Pop.	% pop.	UFP (#·cm^{-3})					$PM_{2.5}$ (μg·m^{-3})				
					Exposure Percentiles									
					10th	25th	50th	75th	90th	10th	25th	50th	75th	90th
**Age (years)**		<15	3406006	17.40	2427	3319	4513	6331	8347	3.59	5.18	8.10	12.08	17.19
		15–44	7833333	40.02	2493	3413	4635	6503	8536	3.62	5.25	8.17	12.18	17.24
		45–64	5186674	26.50	2331	3173	4314	6018	7953	3.56	5.12	8.00	11.90	17.01
		>64	3146306	16.08	2301	3125	4239	5901	7793	3.54	5.11	7.97	11.85	16.91
		Total	*19572319*	*100.00*										
**Race-ethnicity**		Hispanic	3720983	19.01	3195	4439	6195	8805	12001	3.83	5.57	8.69	13.23	18.76
	Non-Hispanic	Asian	1633539	8.35	3390	4722	6588	9377	12580	3.95	5.69	8.83	13.45	19.10
		Black	2790504	14.26	3110	4337	5962	8469	11228	3.82	5.57	8.63	13.05	18.41
		Native	46034	0.24	1991	2592	3498	4736	6165	3.40	4.90	7.64	11.12	15.74
		Other	491758	2.51	2547	3467	4733	6668	8729	3.64	5.28	8.19	12.26	17.27
		Pacific	5689	0.03	2420	3284	4438	6216	8109	3.65	5.24	8.15	12.10	17.18
		White	10883812	55.61	1908	2609	3505	4822	6320	3.37	4.86	7.65	11.14	16.16
		Total	*19572319*	*100.00*										
**Economic status**	Poverty level	Below	2681277	14.07	2604	3547	4848	6780	8903	3.66	5.34	8.29	12.37	17.39
		Above	16381903	85.93	2397	3265	4442	6216	8190	3.58	5.16	8.08	12.01	17.14
		Total	*19063180*	*100.00*										
	House ownership	Owner	3957802	53.90	2026	2726	3703	5085	6639	3.39	4.90	7.68	11.25	16.33
		Renter	3385432	46.10	2856	3960	5443	7672	10102	3.79	5.55	8.56	12.86	17.92
		Total	*7343234*	*100.00*										

	Ratio of income to poverty level	< 0.5	197322	4.26	2590	3532	4837	6749	8877	3.65	5.32	8.24	12.31	17.37
		0.5–1.0	282629	6.10	2683	3660	5020	7038	9218	3.69	5.38	8.32	12.50	17.43
		1.0–1.5	319422	6.90	2550	3468	4760	6639	8704	3.64	5.31	8.22	12.29	17.35
		1.5–2.0	319788	6.90	2426	3293	4482	6262	8206	3.60	5.20	8.11	12.04	17.21
		2.0–3.0	612812	13.23	2279	3075	4170	5802	7602	3.54	5.10	7.97	11.79	16.77
		>3.0	2900316	62.61	2285	3122	4238	5914	7825	3.53	5.08	7.96	11.80	16.89
		Total	*4632289*	*100.00*										
	Income ($)	<10k	482798	6.57	2616	3602	4893	6834	9002	3.68	5.36	8.33	12.41	17.47
		10–20k	669968	9.12	2403	3277	4452	6197	8120	3.59	5.23	8.15	12.06	17.10
		20–30k	590604	8.04	2290	3087	4189	5819	7612	3.53	5.12	8.01	11.82	16.74
		30–40k	559229	7.62	2256	3045	4119	5721	7462	3.52	5.10	7.99	11.78	16.69
		40–50k	510337	6.95	2221	2996	4047	5597	7321	3.50	5.06	7.96	11.72	16.66
		50–75k	1116475	15.20	2232	3017	4080	5647	7383	3.51	5.08	7.96	11.72	16.65
		75–100k	879510	11.98	2266	3062	4151	5784	7566	3.52	5.10	7.96	11.79	16.75
		100–125k	672336	9.16	2304	3139	4260	5931	7811	3.55	5.13	7.98	11.87	16.95
		125–150k	478047	6.51	2372	3246	4415	6175	8141	3.57	5.15	8.06	12.00	17.08
		150–200k	595454	8.11	2507	3449	4702	6630	8753	3.61	5.19	8.14	12.26	17.38
		>200k	788476	10.74	2794	3898	5424	7668	10329	3.73	5.43	8.46	12.85	18.16
		Total	*7343234*	*100.00*										
**Urbanicity**	Metropolitan	Large	10082012	51.51	3558	4875	6781	9573	12508	4.00	5.81	8.92	13.51	18.96
		Large fringe	5446635	27.83	2111	2945	4199	5886	8035	3.24	4.78	7.62	11.79	17.24
		Medium	1825168	9.33	958	1383	1957	2761	3711	2.82	4.33	6.77	10.06	14.39
		Small	846810	4.33	868	1251	1745	2462	3350	2.79	4.23	6.74	10.07	14.52
		Micropolitan	982791	5.02	902	916	1208	1623	2248	3002	2.83	4.17	6.48	9.46
		Noncore	388903	1.99	791	1119	1544	2171	2988	2.68	4.00	6.29	9.34	13.47
		Total	*19572319*	*100.00*										

Table S2. County-level distribution statistics for daily population-weighted UFP and $PM_{2.5}$ exposure in New York State by age, race-ethnicity, economic status indicators, and urbanicity. Percentage of population (%-pop.) is rounded to two decimals. UFP is rounded to whole number and $PM_{2.5}$ is rounded to two decimal places.

			Pop.	% pop.	UFP (#·cm^{-3}) Exposure Percentiles					$PM_{2.5}$ (µg·m^{-3})				
					10th	25th	50th	75th	90th	10th	25th	50th	75th	90th
Age (years)		<15	3406006	17.40	2341	3183	4326	6056	7951	3.57	5.12	8.03	11.92	17.01
		15–44	7833333	40.02	2399	3260	4449	6242	8179	3.60	5.20	8.10	12.06	17.12
		45–64	5186674	26.50	2251	3048	4136	5750	7595	3.52	5.07	7.92	11.75	16.81
		>64	3146306	16.08	2221	3004	4073	5659	7478	3.51	5.05	7.92	11.69	16.74
		Total	*19572319*	*100.00*										
Race-ethnicity		Hispanic	3720983	19.01	3105	4301	6000	8536	11594	3.80	5.53	8.63	13.11	18.65
	Non-Hispanic	Asian	1633539	8.35	3308	4585	6389	9079	12204	3.92	5.65	8.77	13.32	18.95
		Black	2790504	14.26	3008	4173	5742	8139	10830	3.81	5.52	8.57	12.97	18.22
		Native	46034	0.24	1878	2471	3319	4508	5871	3.35	4.83	7.50	10.93	15.52
		Other	491758	2.51	2434	3317	4519	6342	8361	3.62	5.22	8.12	12.12	17.19
		Pacific	5689	0.03	2313	3140	4260	5929	7751	3.61	5.18	8.05	11.97	17.04
		White	10883812	55.61	1822	2487	3346	4595	6055	3.34	4.80	7.53	11.02	15.98
		Total	*19572319*	*100.00*										
Economic status	Poverty level	Below	2681277	14.07	2516	3440	4688	6544	8597	3.64	5.31	8.25	12.29	17.31
		Above	16381903	85.93	2300	3140	4255	5943	7839	3.55	5.10	7.99	11.87	16.97
		Total	*19063180*	*100.00*										
	House ownership	Owner	3957802	53.90	1930	2609	3522	4857	6365	3.37	4.85	7.60	11.13	16.11
		Renter	3385432	46.10	2778	3830	5275	7400	9768	3.76	5.51	8.52	12.76	17.83
		Total	*7343234*	*100.00*										

	Ratio of income to poverty level	< 0.5	197322	4.26	2517	3427	4670	6514	8552	3.63	5.30	8.21	12.26	17.29
		0.5–1.0	282629	6.10	2611	3564	4870	6812	8946	3.68	5.35	8.27	12.44	17.40
		1.0–1.5	319422	6.90	2482	3367	4601	6436	8411	3.63	5.27	8.16	12.21	17.23
		1.5–2.0	319788	6.90	2352	3188	4329	6050	7926	3.59	5.17	8.04	11.95	17.09
		2.0–3.0	612812	13.23	2203	2976	4029	5573	7329	3.51	5.06	7.91	11.68	16.62
		>3.0	2900316	62.61	2191	2982	4045	5633	7436	3.48	5.03	7.87	11.71	16.73
		Total	*4632289*	*100.00*										
	Income ($)	<10k	482798	6.57	2536	3482	4714	6583	8667	3.65	5.33	8.27	12.34	17.35
		10–20k	669968	9.12	2344	3165	4291	5951	7822	3.58	5.21	8.09	11.93	16.92
		20–30k	590604	8.04	2207	2973	4025	5560	7306	3.51	5.08	7.94	11.71	16.61
		30–40k	559229	7.62	2183	2936	3968	5477	7180	3.50	5.07	7.92	11.65	16.56
		40–50k	510337	6.95	2137	2881	3898	5369	7035	3.48	5.03	7.87	11.57	16.46
		50–75k	1116475	15.20	2152	2896	3918	5404	7108	3.48	5.04	7.89	11.60	16.49
		75–100k	879510	11.98	2186	2951	3985	5544	7254	3.50	5.04	7.90	11.65	16.61
		100–125k	672336	9.16	2226	3013	4078	5672	7462	3.51	5.06	7.93	11.72	16.75
		125–150k	478047	6.51	2286	3122	4235	5929	7822	3.54	5.09	7.97	11.86	16.92
		150–200k	595454	8.11	2410	3298	4495	6313	8375	3.57	5.14	8.06	12.07	17.26
		>200k	788476	10.74	2676	3729	5186	7327	9829	3.67	5.37	8.36	12.71	18.00
		Total	*7343234*	*100.00*										
**Urbanicity**	Metro-politan	Large	10082012	51.51	3507	4800	6668	9401	12247	3.99	5.82	8.93	13.48	18.93
		Large fringe	5446635	27.83	1968	2739	3906	5453	7457	3.16	4.67	7.46	11.50	16.91
		Medium	1825168	9.33	876	1256	1766	2492	3384	2.74	4.18	6.58	9.78	14.11
		Small	846810	4.33	838	1195	1678	2375	3219	2.80	4.23	6.66	9.91	14.36
		Micropolitan	982791	5.02	902	1190	1594	2209	2938	2.84	4.16	6.45	9.38	13.42
		Noncore	388903	1.99	785	1108	1534	2160	2975	2.68	3.98	6.27	9.33	13.42
		Total	*19572319*	*100.00*										

Supplementary figures

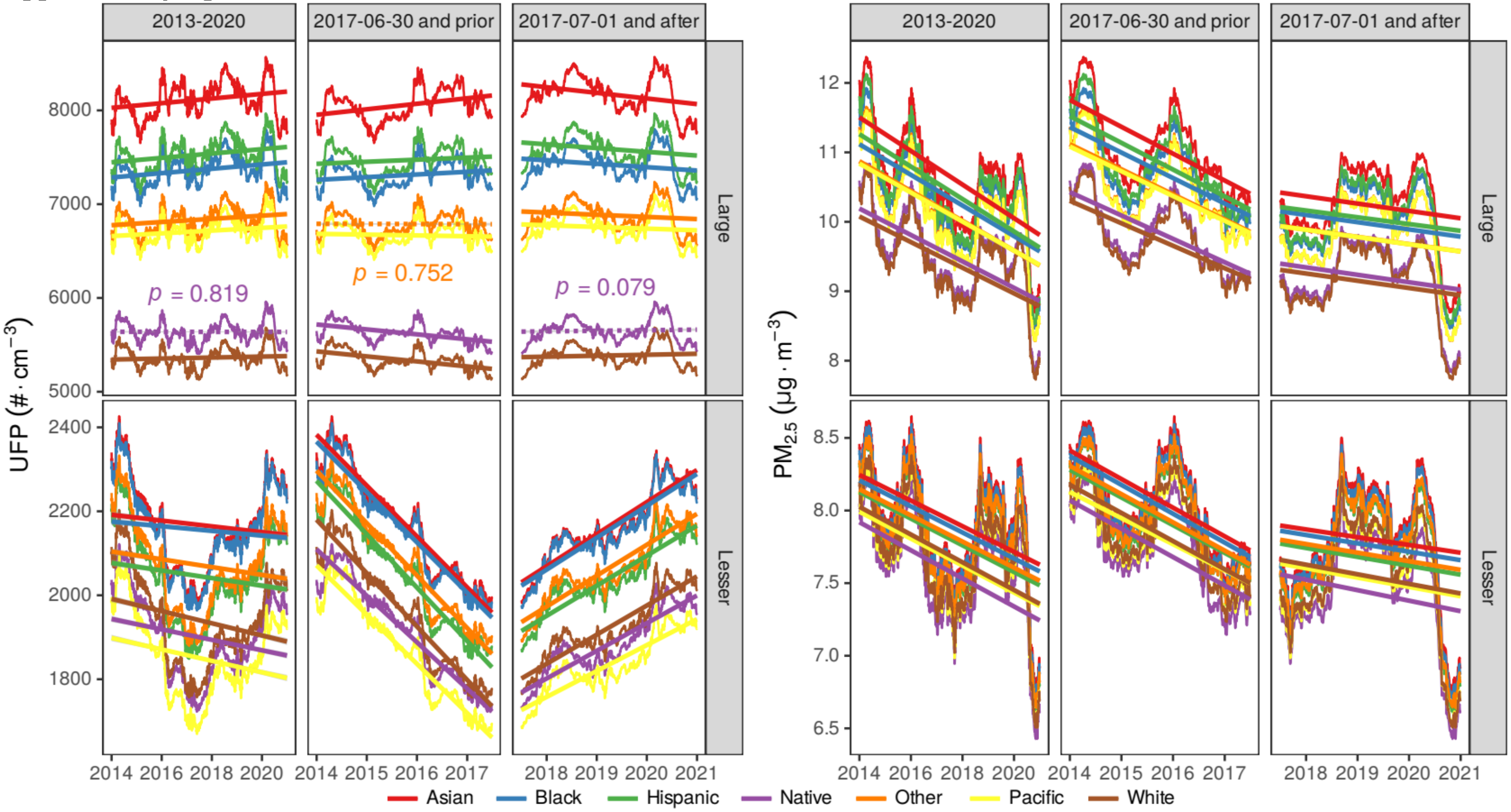

Figure S1. Yearly moving average and linear regression fits for the race-ethnicity group-wise absolute aerosol exposure. The eight-year period of study is divided into two halves. Dichotomized indicator for level of urbanization as Large and Lesser. Large indicates combined large central and large fringe metropolitan areas and Lesser indicates combined medium and small metropolitan, micropolitan, and noncore areas. UFP exposure has been relatively constant in areas of Large urbanicity while in areas of Lesser urbanicity shown an initial decreasing trend followed by increasing trend. $PM_{2.5}$ exposure has been generally declining over the entire period with plateauing of reductions post 2017. Additionally, UFP exposure disparities for minority race-ethnicity groups are much greater than those for $PM_{2.5}$. $p \ll 0.05$ unless indicated. $p \geq 0.05$ indicated with dotted lines.

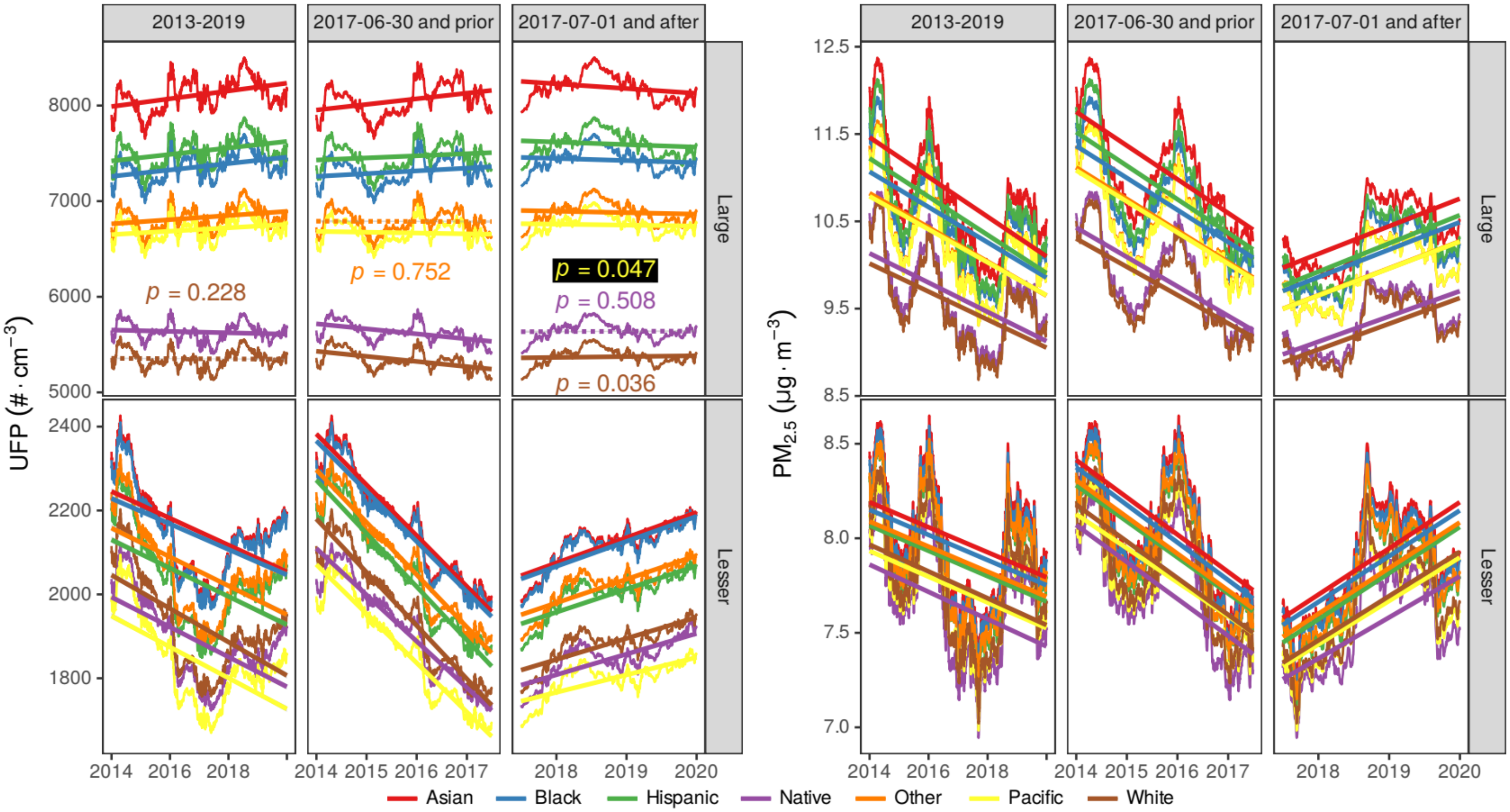

Figure S2. (As Figure S1, minus 2020 to remove the impact of the COVID-19 pandemic period reductions) Yearly moving average and linear regression fits for the race-ethnicity group-wise absolute aerosol exposure. The seven-year period of study is divided at the inflection point of 2017-07-01. Dichotomized indicator for level of urbanization as Large and Lesser. Large indicates combined large central and large fringe metropolitan areas and Lesser indicates combined medium and small metropolitan, micropolitan, and noncore areas. In areas of Large urbanicity, UFP exposure has been relatively constant or slightly increasing while $PM_{2.5}$ exposure has been generally declining over the entire period. Additionally, UFP exposure disparities for minority race-ethnicity groups are much greater than those for $PM_{2.5}$. $p \ll 0.05$ unless indicated. $p \geq 0.05$ indicated with dotted lines.

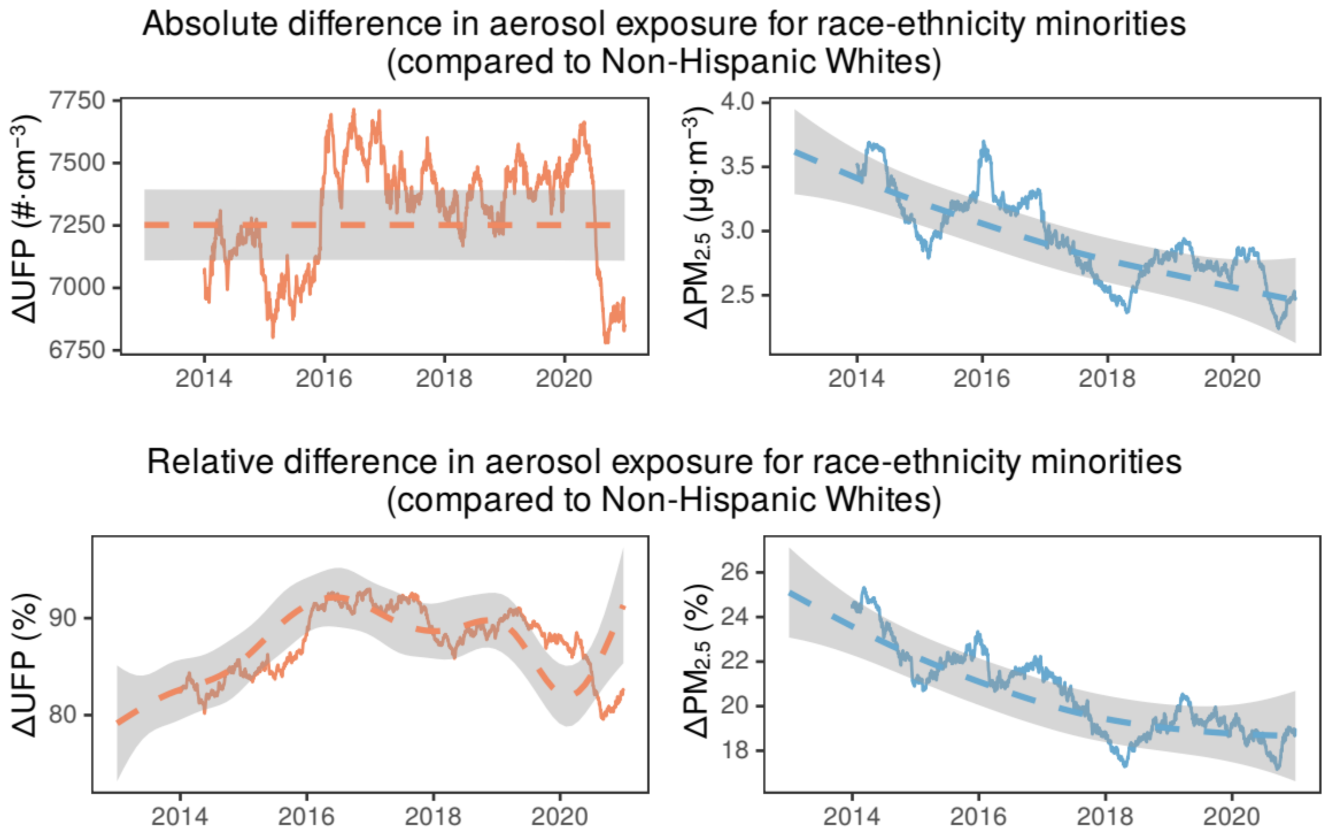

Figure S3. Yearly moving average for the temporal evolution of race-ethnicity disparities in aerosol pollutant high-exposure (>90th percentile hourly for each day) in (top) absolute terms and (bottom) relative terms. Disparities are presented for the aggregated race-ethnicity minority group compared to non-Hispanic White subgroup. Shown on the left (red) are these for UFP^{90} and on the right (blue) for $PM_{2.5}^{90}$. UFP exposure disparities are larger and unabating as compared to those for $PM_{2.5}^{90}$.

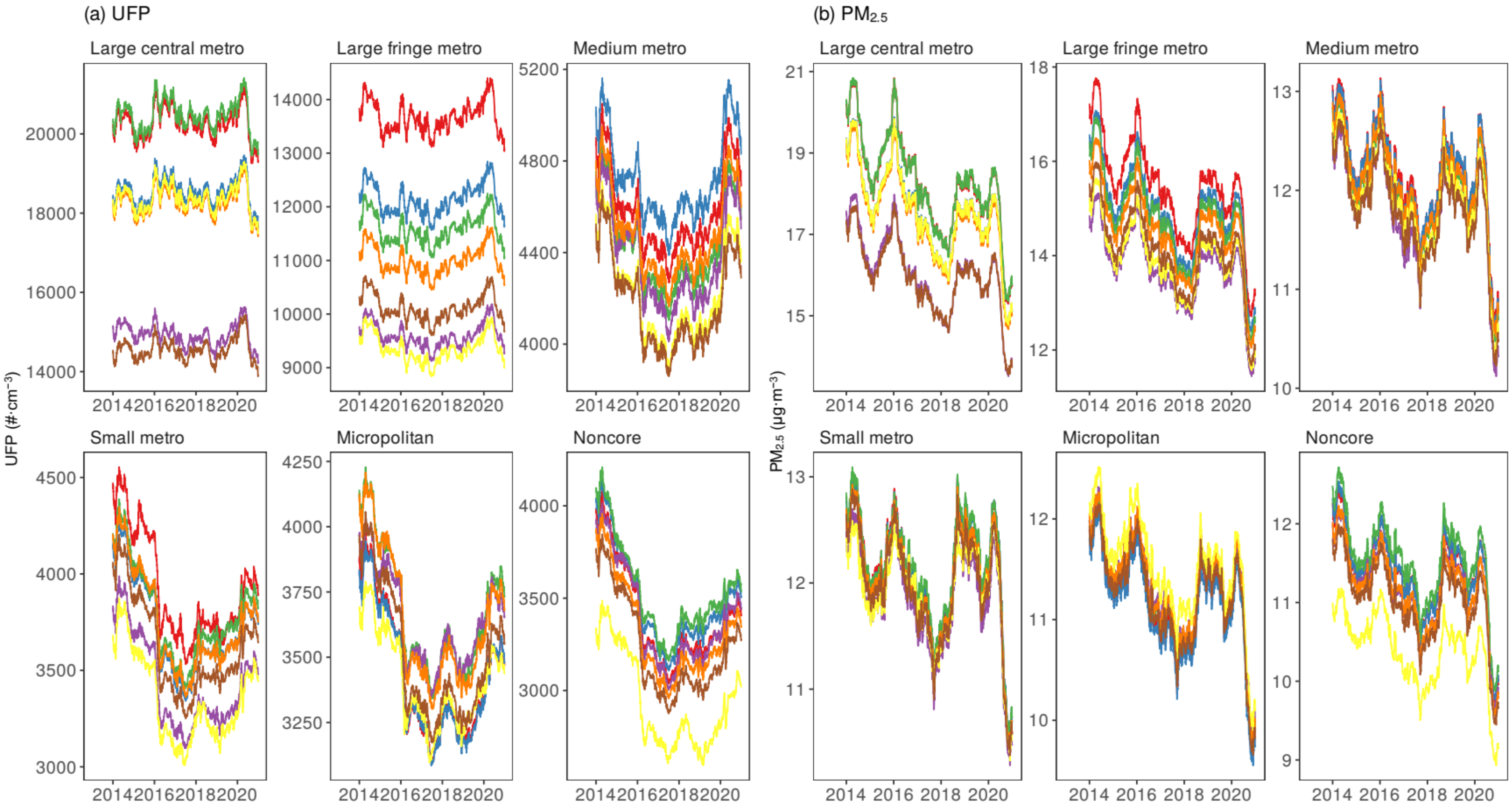

Figure S4. Yearly moving average for (a) UFP^{90} and (b) $PM_{2.5}^{90}$ population-weighted exposure in NYS during 2013–2020. Each race-ethnicity group is indicated by colors: red (Asian), blue (Black), green (Hispanic), purple (Native), orange (Other), yellow (Pacific), brown (White). Plots are faceted by NCHS urban classification areas.

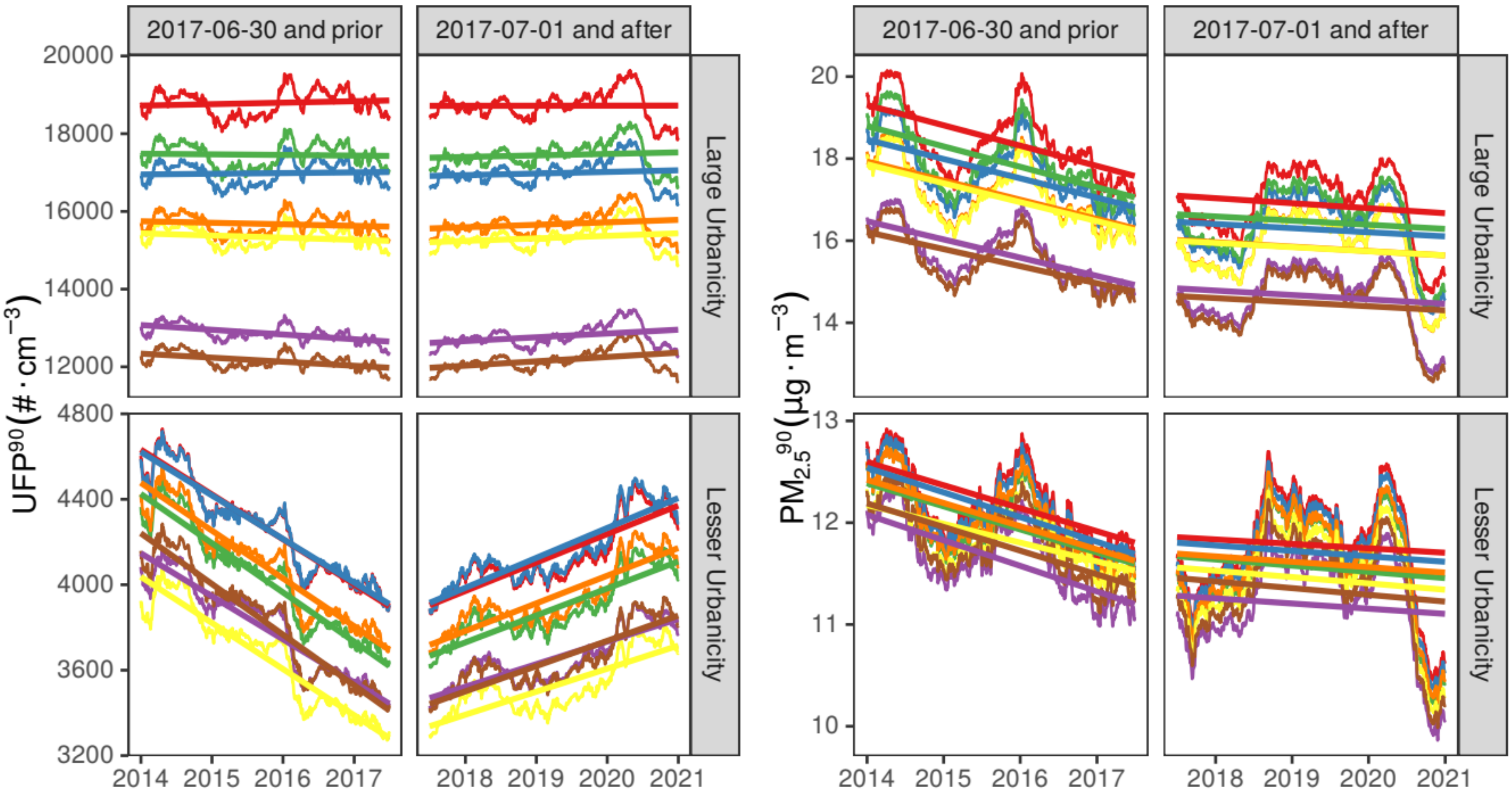

Figure S5. Yearly moving average and linear regression fits for the race-ethnicity group-wise absolute aerosol high-exposure. The eight-year period of study is divided into two halves. Binomial indicator for level of urbanization as Large and Lesser. Large indicates combined large central and large fringe metropolitan areas and Lesser indicates combined medium and small metropolitan, micropolitan, and noncore areas. UFP^{90} exposure has been relatively constant in areas of Large urbanicity while in areas of Lesser urbanicity shown an initial decreasing trend followed by increasing trend. $PM_{2.5}{}^{90}$ exposure has been generally declining over the entire period with plateauing of reductions post 2017. Additionally, UFP^{90} exposure disparities for minority race-ethnicity groups are much greater than those for $PM_{2.5}{}^{90}$.

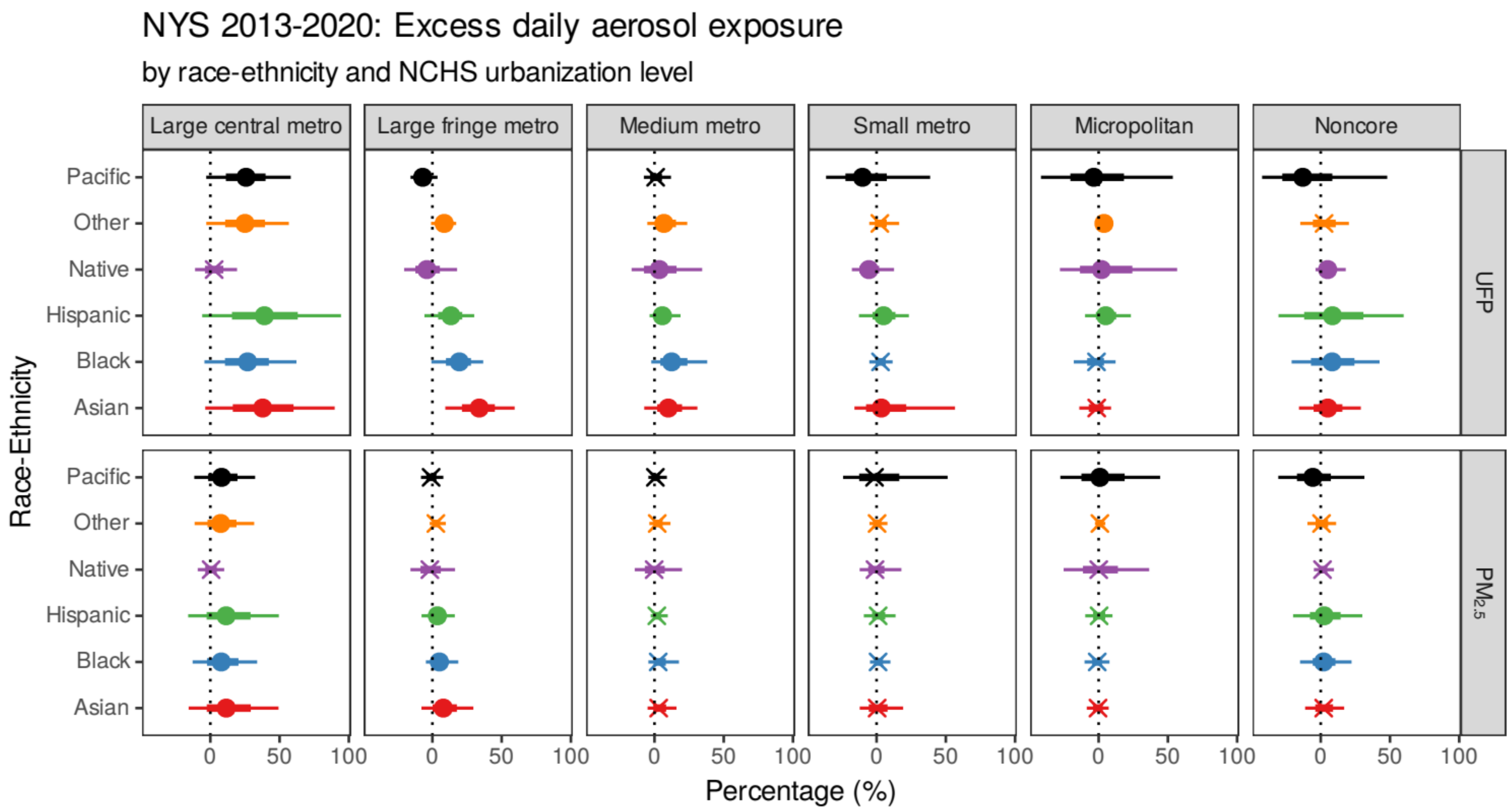

Figure S6. The relative excess exposure (%) to UFP^{90} and $PM_{2.5}^{90}$ for race-ethnicity groups compared to the non-Hispanic White subgroup by urbanicity in New York State. Circles indicate a statistically significant difference between distributions (each vs. non-Hispanic White) and crosses otherwise.

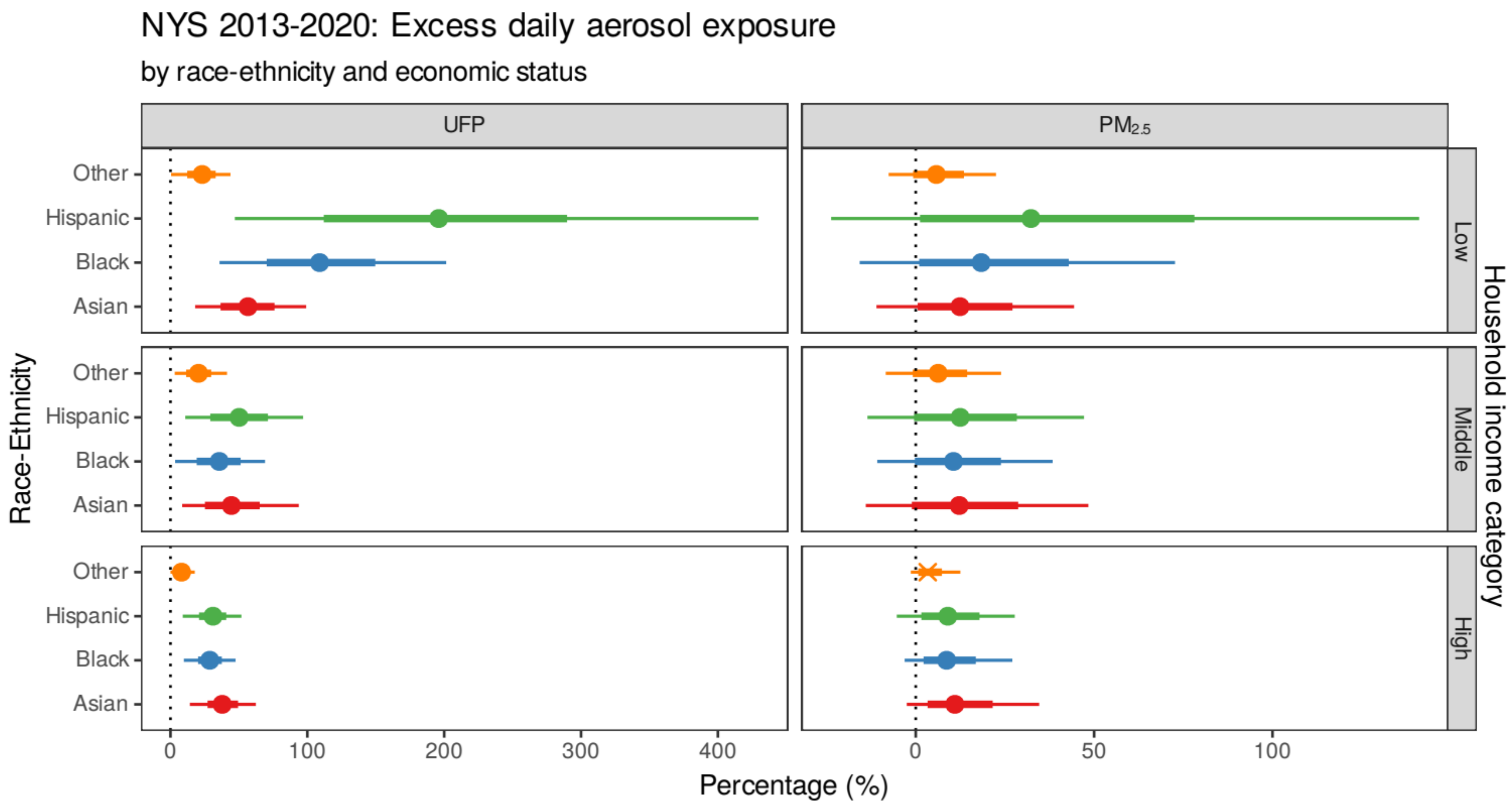

Figure S7. The relative excess exposure (%) to UFP^{90} and $PM_{2.5}^{90}$ for race-ethnicity groups compared to the non-Hispanic White subgroup by economic status in New York State. Circles indicate a statistically significant difference between distributions (each vs. non-Hispanic White of corresponding income category) and crosses otherwise. Note the different y-axis scales for each pollutant.

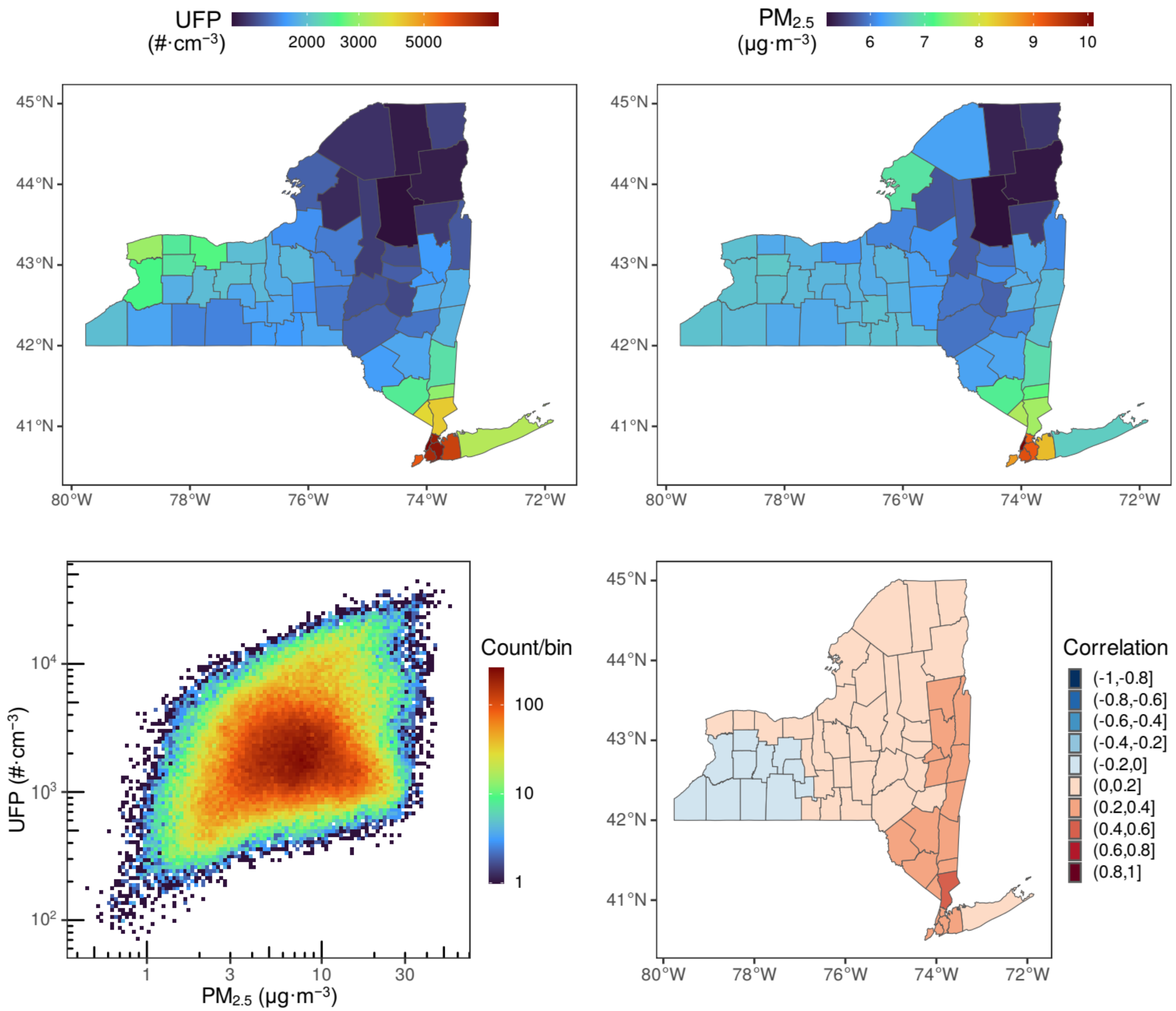

Figure S8. County-level characteristics of ultrafine particle number concentrations (UFP) and $PM_{2.5}$ over New York State (NYS) from 2013–2020. (Top) Spatial distributions of period averaged values for UFP and $PM_{2.5}$. (Bottom) Extent of daily UFP–$PM_{2.5}$ correlation: (left) binned scatter plot and (right) spatially at the county level.

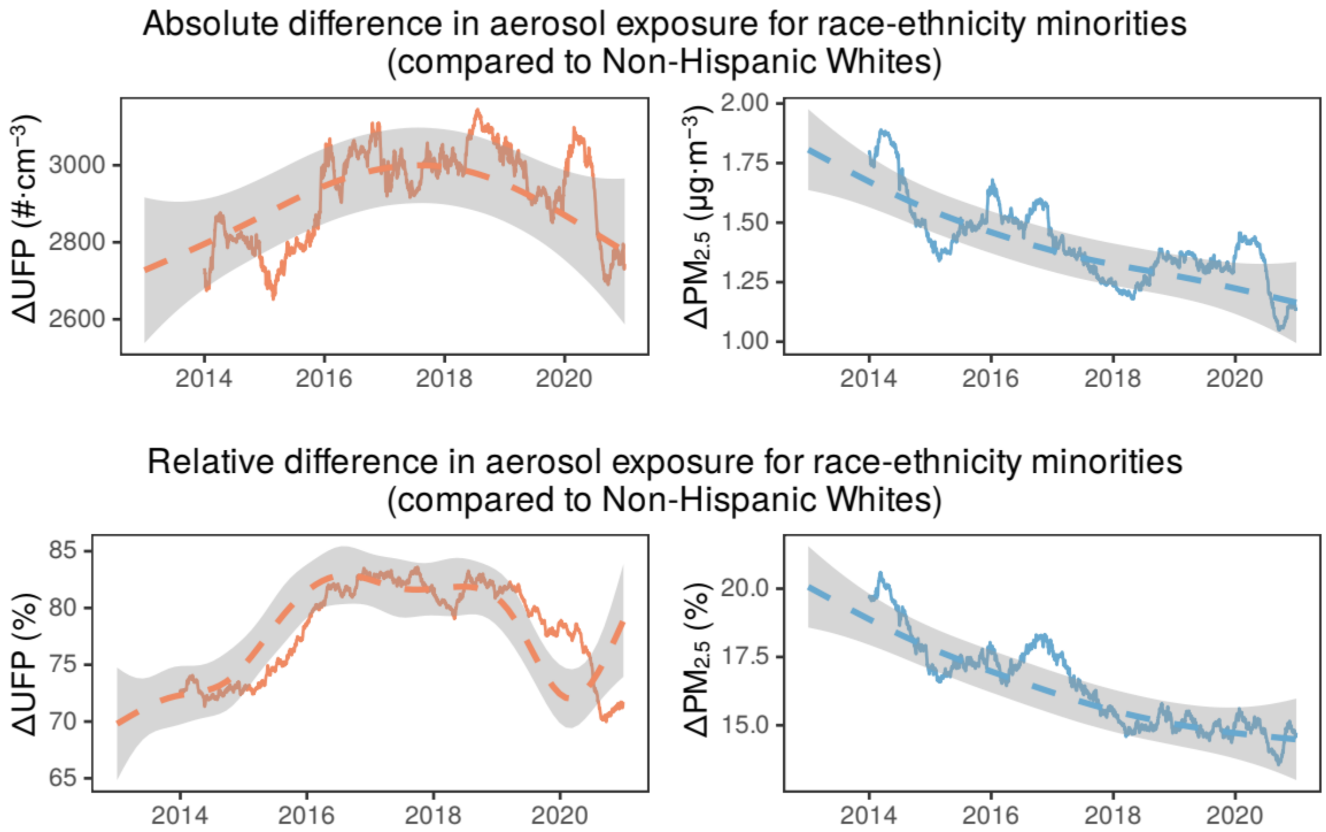

Figure S9. County-level yearly moving average for the temporal evolution of race-ethnicity disparities in aerosol pollutant exposure in (top) absolute terms and (bottom) relative terms. Disparities are presented for the aggregated race-ethnicity minority group compared to non-Hispanic White subgroup. Shown on the left (red) are these for UFP and on the right (blue) for $PM_{2.5}$. UFP exposure disparities are larger and unabating as compared to those for $PM_{2.5}$.

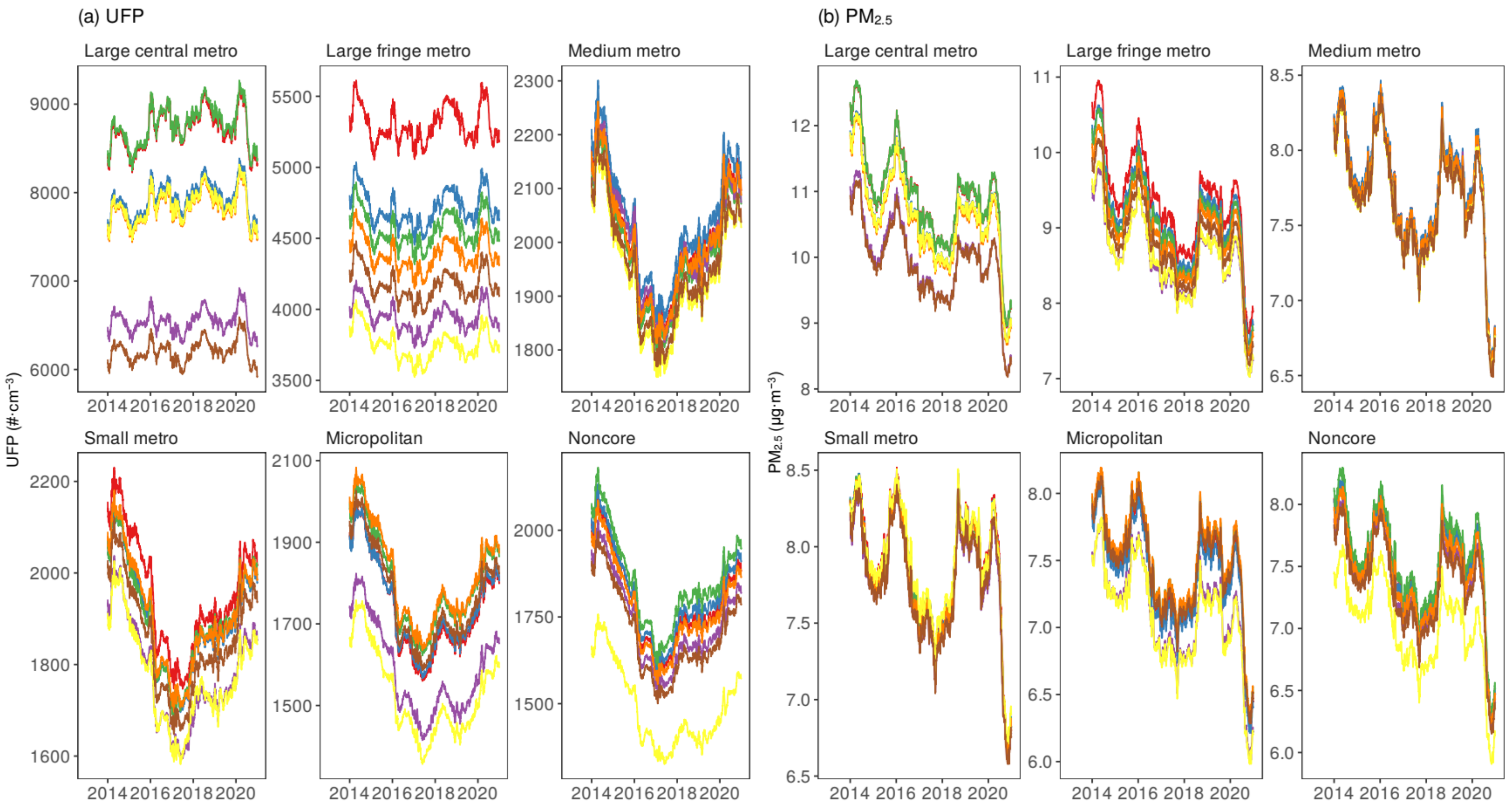

Figure S10. Yearly moving average for (a) UFP and (b) $PM_{2.5}$ population-weighted county-level exposure in NYS during 2013–2020. Each race-ethnicity group is indicated by colors: red (Asian), blue (Black), green (Hispanic), purple (Native), orange (Other), yellow (Pacific), brown (White). Plots are faceted by NCHS urban classification areas.

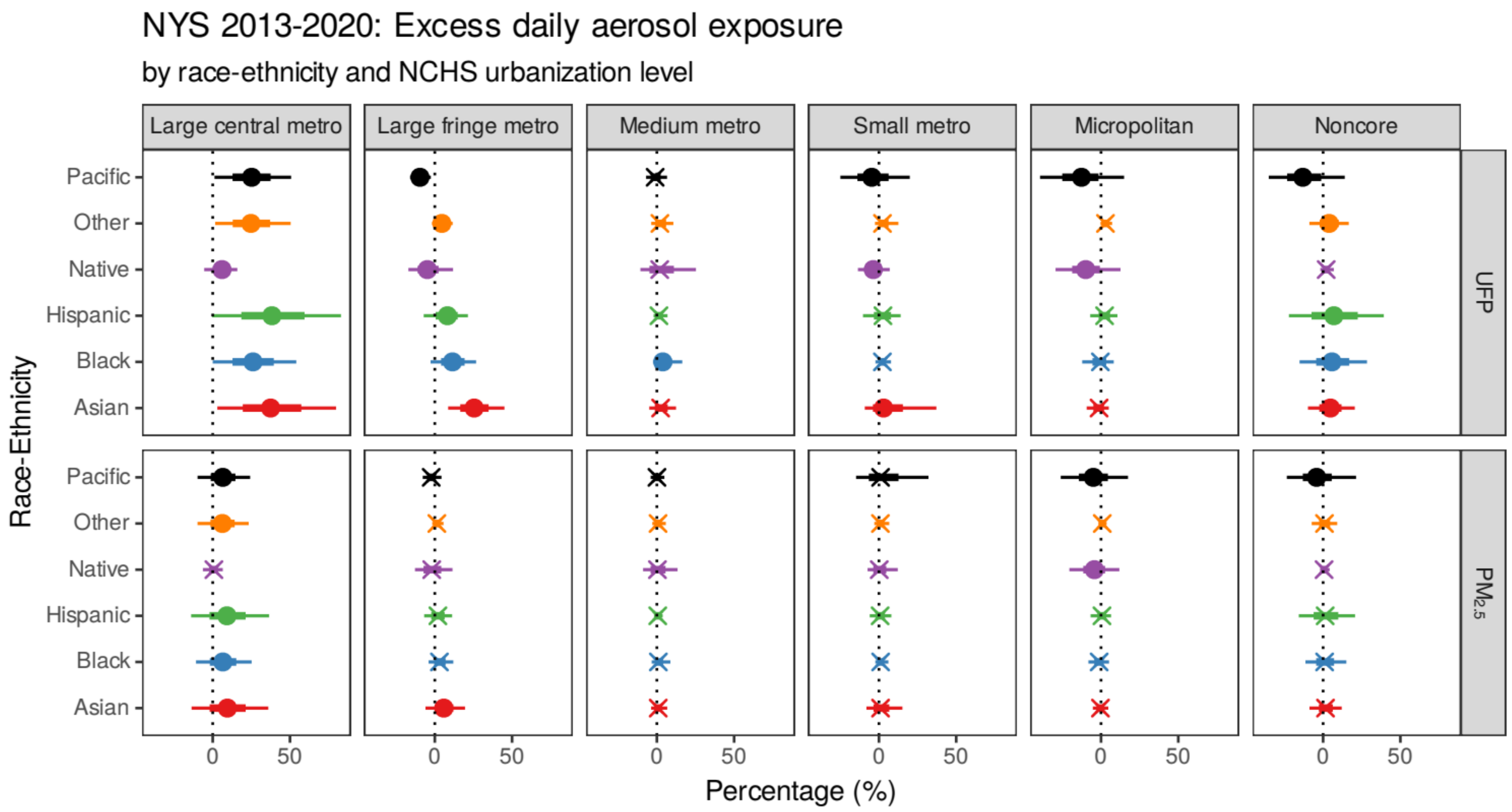

Figure S11. The relative excess exposure (%) to UFP and $PM_{2.5}$ for race-ethnicity groups compared to the non-Hispanic White subgroup by urbanicity in New York State at the county-level. Circles indicate a statistically significant difference between distributions (each vs. non-Hispanic White) and crosses otherwise.
